# Household food insecurity levels in Ethiopia: quantile regression approach

**DOI:** 10.3389/fpubh.2023.1173360

**Published:** 2023-07-10

**Authors:** Habtamu T. Wubetie, Temesgen Zewotir, Aweke A. Mitku, Zelalem G. Dessie

**Affiliations:** ^1^College of Science, Bahir Dar University, Bahir Dar, Ethiopia; ^2^Statistics Department, College of Natural and Computational Science, University of Gondar, Gondar, Ethiopia; ^3^School of Mathematics, Statistics and Computer Science, University of KwaZulu-Natal, Durban, South Africa

**Keywords:** individual-specific effect, panel data, principal component analysis, food insecurity, unobserved heterogeneity

## Abstract

**Introduction:**

Numerous natural and man-made factors have afflicted Ethiopia, and millions of people have experienced food insecurity. The current cut-points of the WFP food consumption score (FCS) have limitations in measuring the food insecurity level of different feeding patterns due to the diversified culture of the society. The aim of this study is to adapt the WFP food security score cut-points corrected for the different feeding cultures of the society using effect-driven quantile clustering.

**Method:**

The 2012, 2014, and 2016 Ethiopian socio-economic household-based panel data set with a sample size of 3,835 households and 42 variables were used. Longitudinal quantile regression with fixed individual-specific location-shift intercept of the free distribution covariance structure was adopted to identify major indicators that can cluster and level quantiles of the FCS.

**Result:**

Household food insecurity is reduced through time across the quintiles of food security score distribution, mainly in the upper quantiles. The leveling based on effect-driven quantile clustering brings 35.5 and 49 as the FCS cut-points corrected for cultural diversity. This corrected FCS brings wider interval for food insecure households with the same interval range for vulnerable households, where the WFP FCS cut-points under estimate it by 7 score. Education level, employment, fertilizer usage, farming type, agricultural package, infrastructure-related factors, and environmental factors are found to be the significant contributing factors to food security. On the other hand, the age of the head of the household, dependency ratio, shock, and no irrigation in households make significant contributions to food insecurity. Moreover, households living in rural areas and farming crops on small lands are comparatively vulnerable and food insecure.

**Conclusion:**

Measuring food insecurity in Ethiopia using the WFP FCS cut-off points underestimates households’ food insecurity levels. Since the WFP FCS cut-points have universality and comparability limitations, there is a need for a universally accepted local threshold, corrected for local factors those resulted in different consumption patterns in the standardization of food security score. Accordingly, the quantile regression approach adjusts the WFP-FCS cut points by adjusting for local situations. Applying WFP cut-points will wrongly assign households on each level, so the proportion of households will be inflated for the security level and underestimated for the insecure level, and the influence of factors can also be wrongly recommended the food security score for the levels. The quantile clustering approach showed that cropping on a small land size would not bring about food security in Ethiopia. This favors the Ethiopian government initiative called integrated farming “ኩታ ገጠም እርሻ” which Ethiopia needs to develop and implement a system that fits and responds to this technology and infrastructure.

## Introduction

Sufficient, safe, and nutritious food availability, access, and utilization for all people at all times are very important hierarchal pillars that ensure household-level food security (1). Food insecure and vulnerable households are those whose food intake is less than the food intake of food-secure households (2). Food insecurity is a global burden on 928 million of the global population in 2020, which is 148 million more than in 2019 (3).

The underlying factors challenging food security and nutrition are mainly conflicts and wars. In 2020, nearly 75% of the world’s stunted children lived in Central and Southern Asia (37%) and sub-Saharan Africa (37%) (3, 4). Drought is the main cause of crop and livestock loss (89%) from climate disasters in Africa (5). In sub-Saharan countries, most of the population are agricultural-dependent and struggle for food; they are severely attacked by drought, internal displacement, conflicts, and desert locust (3, 6, 7) and had a higher rate of hunger as of 2010 (8). Economic slowdowns as a result of trade wars, the Russian - Ukraine war (9, 10), and a global pandemic like COVID-19 (9, 11–15) raise the rate of food insecurity in most countries, especially low-income countries (sub-Saharan Africa) that have higher rates of food insecurity due to income inequality (3, 4, 13, 14). Especially poor’s in developing countries faced saver food insecurity and influenced by instable food supply (12, 16) and socio-economic factors (16–18). Studies in Slovak (19), Afghanistan (20), Malawi (18), and Nigeria (21, 22) showed that the impact predictors of food security depend on the level (quantiles) of households’ food insecurity scores.

In Ethiopia, millions of households suffer from food shortages each year, and the government and aid organizations (FAO and WFP) support food and shelter in response to hunger and natural disasters by direct food supply, creating jobs on the farm, or cash transfer (23, 24). Several studies have indicated factors that affect a household’s food security in Ethiopia (22, 25–27).

Food security measurement is an ongoing problem and different studies have different measurements (22, 28). The FCS was first created by the world food program (WFP) in Southern Africa in 1996 as an alternative (29). FCS is a frequency-weighted diet diversity score multiplied by the relative nutritional importance of different food groups for 7 days of consumption (2, 29–31). The cut points for WFP FCS are 21 and 35, i.e., the household is food insecure if FCS is less than or equal to 21, vulnerable if FCS is between 21.5 and 35, and secure if FCS is greater than or equal to 35.5. However, since the measurement considers the number of times eaten and the nutritional contents of the food, it varies based on the community consumption pattern difference, and WFP suggested an adjustment for the cut points by 7 score (i.e., 27 and 42) for communities that usually (6 or 7 days per week) consume small amounts of sugar and oil (2, 22, 28, 32). However, there is a lack of a universally accepted threshold corrected for other local factors, which results in different consumption patterns in the standardization of food security scores. As literatures also indicated that, the cut points for FCS is an ongoing problem due to local factor like, cultural disparity causing differences in the consumption patterns (30, 31). Recent pieces of literature have suggested alternative cut points to WFP food consumption score cut points; for instance, FAO (31) recommends cut points 45 and 61 for Jordan households, and Baumann et al. (30) used cut points 32 and 43 for the Laos context due to cultural disparities. Since the FCS considers diet diversity (33), classifying the level of food insecurity of sub-national areas by rankings is preferable to the direct score cut-points (34). Baumann et al. suggested further investigations in different cultural settings to get insight into universal threshold considerations of local factors such as the exclusion of small amounts of food items (30).

The response variable “food security score” has a longitudinal nature and may change in shape each time (35–37), and fitting it with a longitudinal model visualizes the evolution of an individual trajectory over time and brings extra information due to the unobserved heterogeneity to the model (35, 37). Unlike the standard regression, the quantile regression does answer the question of how input variables affect the response at different quantiles of the distribution (35, 36, 38–40). Therefore, fitting an extended longitudinal model for quantile regression can help to avoid misleading inferences (38).

As a traditional, historical, and religious country, Ethiopia has a very diverse diet which includes; crops, roots, pulses, fruits, vegetables, meat, fish and other stem foods. Furthermore, in addition to various condiment consumption (like ginger, garlic, butter, cheese, paper & other), a small amount of bread, Enjera, drinks, and other grains are consumed (for instance, bread or Enjera with butter, traditional alcohols (Tela and Areki), roasted barley or maize or beans are eaten at a cultural ceremony, coffee ceremony, and religious events such as “Edir,” “Mahiber,” and “Arba/Ametat”). Due to the unique nature of Ethiopian diets, a tailored food consumption assessment is needed, and instead of directly applying the WFP FCS cut points, a flexible approach relative to the population is needed to overcome the shortcomings due to the differences in the dieting culture from community to community (30, 41–43). With this universality and comparability limitation of the WFP FCS cut points, making a food security assessment for a multicultural country like Ethiopia is misleading. Therefore, we plan to adopt an approach that is responsive to the Ethiopian context and compare it with the WFP FCS cut points; it needs to show the gaps in the food insecurity levels within the country and help to know the factors that lead to each level of food insecurity for monitoring and mitigation to reach an interesting level of food security based on the country’s resources. Hence, we proposed the effect-driven leveling approach with the assumption that if some sequence of the quintiles of the FCS (i.e., insecure, vulnerable, and secure) share the same factors (i.e., largely and significantly), those quantiles can be considered as one level and a cut point is fixed based on the quantile interval.

This study is aimed to address the issue with the WFP FCS cut-points by adjusting for different food consumption patterns due to the diversified culture of the society by identifying major indicators that can cluster quantiles of the FCS, which considers the evolutional variability (sustainability over time) of the food security score. Therefore, we adopted an approach by conditioning quintiles of the longitudinal households’ food security scores on causal factors and grouped household scores as one food security score level that shares common major causing factors. Furthermore, we checked these quantiles clustering by using the principal component analysis of the FCS quantiles after coding zero and one for insignificant and significant effects of factors, respectively. Because these clusters of quintiles share some common significantly affecting factors, they should contribute largely to a principal component representing the food security score level. These factors are input for leveling FCS, and monitoring based on those factors can enhance the likelihood of controlling food insecurity for public health improvement beyond the uncertainty of physical phenomena not included in the model. The longitudinal nature of this data can help to find out the evolutional effect of driving factors on households’ food insecurity levels, and the statistical modeling of FCS using those input driving factor values can bring an approximate to each level and do more precise prediction for the future. Focusing on food insecurity reduction brings an improvement in public health because as a frequently drought-affected and unstable low-income country, the resulting food insecurity directly impacted public health in Ethiopia through newborns’ birthweight, stunted and wasted children, and women with anemia (11). Therefore, policymakers and researchers should give attention to measuring and combating food insecurity.

## Methods

### Data

This study analyzed household-based panel data for 3 years (2012, 2014, and 2016) covering the whole region of the country. This panel data recorded households’ weekly (7 days) food consumption and other related factors repeatedly three times. A total sample size of 11,505 (3,835 households with three replications for the years 2012, 2014, and 2016) was taken from the Ethiopian Socioeconomic Survey (ESS) of the World Bank data set, which is the first panel data in Ethiopia collected by a project of the World Bank and central statistical agency (CSA) of Ethiopia to quantify household-level food security and related factors in rural and urban (small and medium town) areas. The ESS sample is a two-stage probability sample. The first stage of the sampling is selecting enumeration areas using simple random sampling from the sample of the Annual Agricultural Sample Survey (AgSS) enumeration areas (EAs). The AgSS EAs were selected based on probability proportional to the size of the population (PPS). The second stage is selecting households for the first survey by simple random sampling from the enumeration areas, but the 2nd and 3rd surveys will collect the data repeatedly from those selected households. The original data set used in this study was taken using this URL link.^1^

### Variable

The response variable of this study is the food security score calculated based on the FAO (2016) FCS formula for 7 days of food consumption recorded from households at the enumeration area level (32). A principal component analysis (PCA) is used to reduce the dimension of the data by merging predictors based on natural relations through a few uncorrelated latent variables without losing much information, each of which is a linear combination of the original variables that can maximize the variance accounted for (44). The principal component analysis was performed as a variable reduction method for Agricultural, Geographic, and Assets factors, and for clustering quantiles of food security score. The components are taken by considering the Eigenvalue (>1), the proportion of variance explained from the total variance, and the subjective meaning of highly contributing components (44, 45). After dimension reduction and exploratory analysis, a total of 42 explanatory variables (x’s) are analyzed (The list of all 42 variables is given in Appendix Table 1).

### Model

Repeatedly taken measurements from a household are correlated and the assumption of traditional regression (constant variance and independent error) fails to fit the modeling procedure, which leads us to consider a longitudinal quantile mixed model instead of other models like time series analysis due to a larger number of subject/households and smaller repeated measurements per subject (46, 47). Accordingly, this study applied a longitudinal conditional quantile regression model to detect and control the unobserved heterogeneity that affects dependency between observations of repeated measures from the same subject to visualize the evolutional variability of the quantiles of household food security scores for the causal effect of those subject predictors. The linear quantile mixed effect model package (*lqmm*) in R-software was used for the analysis (48–50). The longitudinal data in quantile regression can be fitted by a marginal or conditional model. Since our data has a longitudinal nature, conditional quantile regression is appropriate (38).

The proposed model considers individual-specific parameters to account for dependence between longitudinal data, and conditional quantiles are estimated simultaneously by minimizing a weighted piecewise linear quantile loss function. Based on the distribution of the individual-specific parameter, conditional quantile regression has used two modeling approaches: the distribution-free and likelihood-based methods. A distribution-free approach considers a fixed individual-specific intercept and is treated as pure location shift parameters common to all conditional quantiles. This implies that the conditional distribution for each individual has the same shape but different locations as long as the individual-specific effects are different (51, 52).

In the likelihood approach, individual-specific parameters, $\gamma_{i}s,$ are assumed to be independent and identically distributed random variables; the corresponding distribution allows us to explain differences in the response quantiles across individuals or showed a distributional shift for each individual (53). The longitudinal data considered by this study have a small number of repeated measures, and it is not able to reflect a distributional shift and may bring biased estimates for coefficients; however, it can better show a fixed individual-specific location-shift effect (51, 52). Therefore, the fixed individual-specific intercepts are considered and treated as pure location shift parameters (distribution-free) specific to a quantile being estimated. In modeling the random effect, the Gauss-Hermite quadrature allows for all types of covariance matrix implemented in *lqmm*; therefore, the random effect is taken as Gaussian random effects (i.e., Gauss-Hermite quadrature).

The conditional τ – quantile of $y_{it}$ (food security score for t^th^ repeated measure of the i^th^ individual) denoted by $Q_{\tau }\left(y_{it}|\beta \left(\tau \right)\mathrm{|,}\;\gamma_{i},\;x_{i,t}\;\right)$ is given by Equation (1) as follows:


(1) $$Q_{\tau }\left(y_{it}|\beta \left(\tau \right)\;,\;\gamma_{i},\;x_{i,t}\right)=\gamma_{i}\left(\tau \right)+x_{i,t}^{\prime }\;\beta \left(\tau \right)\;$$

For a realization of τ^th^ quantile of $y_{i,t}$ Equation (1) can be given as:

(2) $$y_{i,t}=\gamma_{i}\left(\tau \right)+x_{i,t}^{\prime }\;\beta \left(\tau \right)+\varepsilon_{i,t}\mathrm{in matrix form y}=\gamma \left(\tau \right)+X\beta \left(\tau \right)+\varepsilon \;$$

where τ∁(0,1) , $\varepsilon \sim N\left(0,\sigma^{2}\right)$ is an error term whose τ^th^ conditional quantile is identically null, that is, $Q_{\tau }\left(\varepsilon_{i,t}|\beta \left(\tau \right),\;\gamma_{i},\;x_{i,t}\right)=0$ , or equivalent to the conditional quantile restriction:


(3) $$P\left(\varepsilon_{i,t}\left(\tau \;\right)\leq 0|\beta \left(\tau \right),\gamma_{i},x_{i,t}\right)=\tau \;$$

while β(τ) summarizes the effect of the covariates $x_{i,t}$ on the i^th^ household’s food security score, $\gamma_{i}\left(\tau \right)\;$ individual specific variability/effect, and the τ^th^ response quantile for a subject whose baseline level is equal to $\gamma_{i}\left(\tau \right)\;$ ; conditional on $\gamma_{i}\left(\tau \right)\;$ , repeated measures are no longer dependent. The degree of unobserved heterogeneity is characterized by τ-specific variance parameters $\gamma_{i}\left(\tau \right)\;:$ $\gamma_{i}\sim N\left(0,\sigma_{\gamma_{i}}^{2}\right)$ . The $\gamma_{i}\left(\tau \right)\;$ has a pure location shift effect on the conditional τ -quantiles of the response (50, 51).

The method of removing unobservable heterogeneity by differencing or other transformations does not work in longitudinal/panel quantile regression models as regression models. For example on the differencing: $y_{i,t}-y_{i,t-1}$ $=(x_{i,t}$ - $x_{i,t-1}{)}^{\prime }\beta_{0}\;\left(t\right)+\begin{array}{c} \underset{\_\_\_\_\_\_\_\_\_\_\_\_\_\_\_\_\_}{\varepsilon_{i,t}\left(t\right)-\varepsilon_{i,t-1}\left(t\right)}\\ =v_{i,t}\left(t\right) \end{array}$ , $v_{i,t}\left(t\right)$ does not satisfy the desired conditional quantile restriction (3) (49, 50, 54, 55).

In this study food security assessment applied is in a perspective of “effect driven leveling of FCS” governed for difference food pattern due to Ethiopia’s cultural diversity, with cut-points fixed by clustering those conditional quantiles of FCS shared some common major causing factors as one level. Furthermore, we have checked these quantiles clustering by using the principal component analysis of the FCS quantiles after coding zero and one for insignificant and significant effects of factors, respectively. Because these clusters of quintiles shared some common significantly affecting factors, they should contribute largely to a principal component representing that level.

## Results

From exploratory analysis, the principal component analysis reduces the dimension of geographic variables from 19 to six components with an Eigenvalue greater than one which explains 76.12% of the total variation; similarly, 12 agricultural variables combined into four components with an Eigenvalue greater than one explaining 53% of the total variation, and 47 assets variables merged into 12 components with an Eigenvalue greater than one explaining 50.11% of the total variation.

The descriptive result in Table 1 indicates that the food security score has improved over time over the quantiles. The mean approximates the median, and other quantiles (25 Vs 75 and 10 Vs 90) are approximately at an equal distance from the median. The longitudinal quantile regression given by Equation (1) has better precision (smaller standard error) compared to the linear mixed model (Appendix Table 1) and linear quantile regression estimates (Appendix Table 2), with more significant variables. In addition, the tails of the quantile plot suggested the presence of heterogeneous variance on lower and upper quantiles (Appendix Figure 3). Therefore, the suitable model is longitudinal quantile regression with the free distribution assumption of covariance structure in which the individual-specific intercept is just a location shift for each individual. Although for these three-time replications, the covariance structure has to be modeled by distribution-free, an alternative modeling by different models was tried and a convergence criterion was not met.

**Table 1:** Quantiles of FCS for the years 2012, 2014, and 2016, and longitudinal data of 2012–2014-2016.

Year	q0.1	q0.25	q0.35	q0.5	q0.75	q0.9	Mean
2012	23	35	39	47	60.5	76	48.41
2014	24.5	36.5	42	49	63	76.8	50.59
2016	26.5	37	42	49.5	63	77	50.94
Longitudinal	24.5	35.5	41.5	49	62.5	76.5	49.98

The key result for the longitudinal quantile model is given in Table 2 (the full result is given in Appendix Table 3). The major effects of the conditional quantiles’ distribution of food security score suggested three clusters of quantiles which leveled the FCS into three clusters with approximate cut points at the 25th and 50th quantiles. Since each cluster of quantiles of the FCS is dominantly influenced by some significant effects, through coding significant effects by one and zero for less influencing (statistically non-significant) effects, we can strengthen the suggestion of effect-driven clustering for households’ food security score. Accordingly, the principal component analysis result in Appendix Table 4 based on the significance of major effects given in Appendix Table 5 comes with the same cut points as the above-suggested three clusters of quantiles of food security score. Specifically, the cut points are 35.5 and 49, and using these cut points, the food insecure, vulnerable, and secure households are 25, 27.1, and 47.9% of the total household population. This indicates that correcting the WFP FCS cut-points based on leveling the FCS using effect-driven quantile clustering governed for Ethiopia’s cultural diversity has an effect on feeding patterns and brings a wider interval by a score of seven for insecure households with larger proportions, but the interval for vulnerable households is equivalent compared to WFP cut-points 28 and 42.

**Table 2:** Longitudinal quantile regression and linear mixed model (LMM) results for factors that have significant and larger effects.

Estimates	q0.10	q0.25	q0.35	q0.5	q0.75	q0.9
Intercept	−1421.9***	−1432.5***	−1438.5***	−1439.1***	−1440***	−1425.6***
Year (x_1_)	0.72***	0.74***	0.74***	0.74***	0.74***	0.74***
Urban *Vs* Rural (x_3_)	4.23	0.85	0.27	2.06	4.09***	4.6**
Read and write (x_5_)	0.65	0.55	−1.98*	1.98***	2.59***	3.41***
Shock (x_6_)	−3.33***	−1.43*	−0.58	−1.89***	−0.98*	−0.66
Fertilizer (x_7_)	−0.65	−0.18	1.44**	0.2	2.28***	2.24**
Adult equivalence (x_8_)	−1.45	−1.48**	−0.8	−1.31**	−0.99*	−1.07**
Age of household head (x_9_)	−0.08*	−0.07***	−0.03*	−0.01	−0.02	0
Coping strategy index (x_11_)	−0.12	−0.14**	−0.12***	−0.06**	−0.05	−0.05*
Dependency ratio (x_12_)	−1.22**	−1.05***	−0.71***	−0.45**	−0.1	−0.16
Employed (x_14_)	3.38	3.14**	3.21***	3.91***	4.65***	5.35***
Farm type (x_15_): [Livestock]	4.65	5.9**	6.44***	8.82***	8.87***	6.6**
Farm type (x_15_): [Both farms]	−0.79	1.33	1.07	1.77***	4.24***	2.88*
Health problem (x_18_)	−0.63	−1.79*	−2.19**	−1.81***	−0.64	−0.55
Household size (x_19_)	1.78**	1.39***	1.04*	1.5***	1.29***	1.6***
Small-size land ownership (x_20_)	−3.86*	−3.78***	−2.74**	−2.61***	−0.96	−2.17
Soil property related (x_24_)	0.75	0.85*	0.64*	0.4	1.44**	1.49**
Agro-ecological and distance from border-related (x_25_)	0.25	0.71	0.24	−0.91***	−0.95**	−0.52
Rainfall and greens related (x_26_)	−0.71	−1.4**	−0.86**	−1.15***	−1.18**	−1.77**
Agricultural package related (x_30_)	1.14	1.41***	0.44	1.08***	0.7*	0.65*
Drinking water (x_33_)	−0.83	−0.09	0	0.41	0.15	0.47
Irrigation-related (x_37_)	0.74	1.67***	0.01	−0.15	−0.15	−0.48
Non-agricultural business related (x_38_)	0.54	0.67	0.78***	0.9***	0.84**	0.47
Sanitation-related (x_41_)	0.57	0.72*	1.19***	1.32***	1.43***	1.55***
AIC	100,833	99,616	99,189	98,979	100,717	102,742
Log-likelihood	−50,362	−49,753	−49,539	−49,434	−50,304	−51,316
The covariance matrix of the random effects: (Individual-specific variability, $\sigma_{\gamma_{i}}^{2}$ )	105.9	108.3	81.44	70.14	108.9	165.7
Residual scale parameter: (standard deviation, $\sqrt{\;\sigma^{2}}$ )	2.087 (21)	4.397 (18.54)	5.391 (17.49)	5.959 (16.85)	4.624 (19.5)	2.205 (22.18)
ICC= $\frac{\sigma_{\gamma_{i}}^{2}}{\sigma_{\gamma_{i}}^{2}+\sigma^{2}}$	0.19	0.24	0.21	0.20	0.22	0.25

Significances: ***for 99%, **for 95% & *for 90%.

The results in Table 2 reveal that the household-specific variability across time on the model is high on the food insecure and secure compared to the vulnerable, with a random effect covariance matrix ranging from 81.44 to 190.6. A smaller intra-correlation coefficient (ICC) ranging from 0.19 to 0.25 is observed over quantiles of food security scores. It is an indication of a low correlation between any two repeated measures within the subject. In general, even if households’ FCS showed smaller progress across all quantiles, better change has been shown in households in food-secure households.

**Figure 1:**
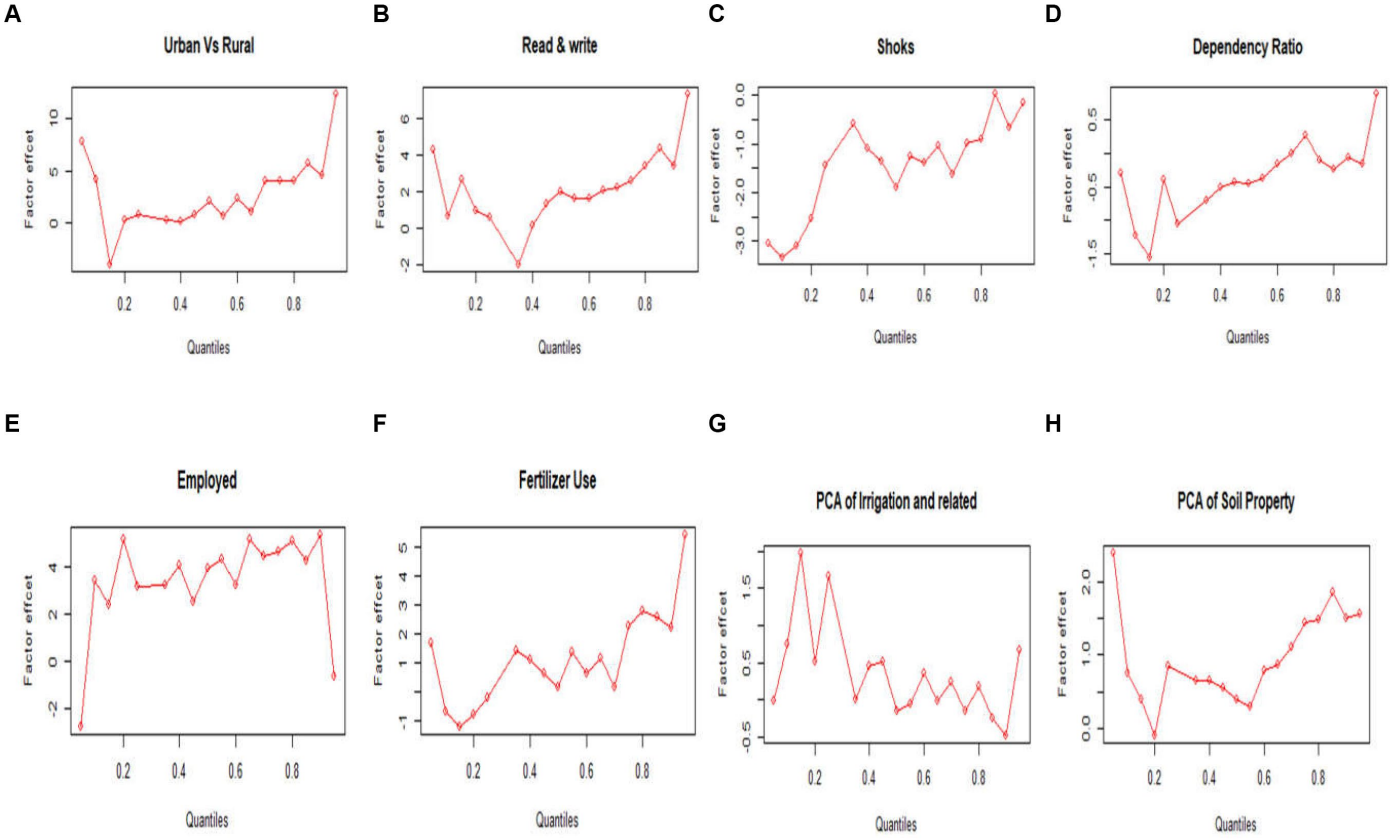
The distributional plot of longitudinal quantile regression coefficients estimate or effect on households’ FCS: **(A)** Urban *Vs* rural, **(B)** Can read and write (yes/no), **(C)** Shock occurred (yes/no), **(D)** Dependency ratio, **(E)** Employed (yes/no), **(F)** Fertilizer used (yes/no), **(G)** Component of irrigation-related, and **(H)** Component of soil quality.

The results in Table 2 and Figure 1 (with Appendix Figures 1, 2) indicate that relative to rural households, urban households have significantly higher FCS mainly in insecure and secure households; the gap rise from 4 to 12 times in secure households and four to 8 in insecure households. Food security score also differs across regional states. Education has a stronger positive significant effect on higher FCS quantiles of households who can read and write compared to those who cannot, with an effect ranging from 1.98 to 7.31. Households led by younger heads are food insecure, and food security increases with the age of households’ heads, ranging from −0.19 (−0.31 — -0.06) to 0.02 (−0.06 — 0.09). Male-headed households are more food secure across the quantiles. Household size has a significant positive effect across all levels of the food security score distribution, but this effect is higher on insecure households, and it rises from 1.78 to 2.07.

Employed-headed households have better food security, and its effect is larger on higher quantiles; its effect increases as quantiles of food security increase, from 3.14 (0.76 — 5.52) to 5.35 (1.75 — 8.96). The component score for non-agricultural business-related has a significant positive effect on the middle quantiles, and its effect declines to the ends of the quantiles of the food security score.

The dependency ratio and shock that occurred have a higher significant negative effect on food-insecure households, and its effect decreased in vulnerable households and became insignificant in food-secure households. Adult equivalence has a proportionally constant negative effect across the quantiles of the food security score. On the other side, the sanitation component score (such as solid waste disposal and bathing and toilet facilities) and getting drinking water have an increasingly positive effect as the quantile of food security scores rises. Households getting health assistance have an increasingly positive effect as the quantiles of FCS rise, whereas facing health problems has a negative higher effect on middle quantiles of food security score relative to the end.

Soil property component score (soil nutrient content and irrigation, oxygen availability, excess salt, and toxicity) or good soil quality has increased food security, mainly on the lower and upper quantiles. The component score for agro-ecological zone, T^o^, and Elevation and Distance from the border and the component score for rainfall and greens have a significantly higher effect on the higher quantiles, and its effect decreases on the lower quantiles. The component score of irrigation, mixed cropping, crop damage, and erosion has a significant effect on the 15th and 25th quantiles of the food security score.

The agricultural package component score such as advisory service, extension program, credit for agriculture, and crop rotation has a significantly higher effect on the middle and higher quantiles of FCS. Farming-type livestock or livestock with cropping and fertilizer usage has an increasingly positive effect as the quantile of food security scores rises. Having a small-size land (either owned or rented) for cropping has a higher negative effect on quantiles of food security score except on higher quantiles. The severe/higher copping strategies are significantly reducing the middle quantiles food security score. The component scores of information source, housing quality, and electronic and furniture-related have an increasingly significant positive impact across the quantiles of the food security score distribution.

## Discussion

Cultural diversity across the globe brings different patterns to feeding culture. The relevance of the assessment and comparison of households’ food insecurity based on weekly diet-dish data for households with different feeding pattern is questionable, and therefore society’s cultural diversity effect on feeding patterns need to be considered. Therefore, there is a need for a universally accepted threshold that is corrected for local factors for those resulting in different consumption patterns in the standardization of food security score. Accordingly, the regression approaches are proposed to adjust the WFP-FCS cut points by adjusting for local situations through driving factors.

Standard regression found the marginal causes of the response at the mean, but it cannot answer the question of how input variables affect the response at different parts of the distribution. Rather, quantile regression can help to assess this effect. Longitudinal quantile regression is an appropriate model when the interest is on the upper or/and lower quantiles of the distribution for repeatedly taken measurements (35, 37, 48, 50, 51, 54, 56, 57). The longitudinal quantile regression model with a fixed individual-specific intercept with free distribution covariance structure results was selected for fitting repeated measures in finding out the exact effect of the factors compared to linear quantile regression and linear mixed model. In the analysis, the fitting of the alternative likelihood approach for the error covariance model with different covariance structures did not converge.

Based on the evidence from the major contribution of the above factors over the quantiles of FCS, the food security score can be categorized into three classes; the food security score less than the 25th quantile (FCS ≤ 35.5) is food insecure, the food security score between the 25th and 50th quantile (35.5 ≤ FCS ≤ 49) is vulnerable to food insecurity, and a score greater than the 50th quantile (FCS ≥ 49) of food security score is food secure. Using the effect-driven leveling cut points, 35.5 and 45, the coverage of food insecure, vulnerable, and secure households is 25, 27.1, and 47.9% of the total household population. The statistical approach using principal component analysis for clustering quantiles by these major effects also gives the same cut point for these three classes, which strengthens the effect-driven clustering of quantiles into three levels.

The yearly quantiles of 2012, 2014, and 2016 indicated an increasing pattern of the food security score across time even if the change is smaller. Even though the individual-specific variability over time is high in food-insecure and secure households compared to vulnerable households, the progress in food-secure households is better. The reduction in food insecurity through time was also indicated by previous studies (19, 22).

Urban area households have significantly higher food security compared to rural households. This result aligns with the Ethiopian government’s plan for mechanized farming and industrial parks to create jobs mostly for employees from rural areas, and it is also supported by previous studies (19, 22, 58). Similarly, food security score differs over the regions across quantiles; specifically, Deredwa and Gambella have better food security across quantiles, and the southern nation and nationality people (SNNP) have a lower food security distribution. Literature also supports the presence of regional food insecurity variation (59–61).

The household head who can read and write has a stronger positive significant effect on food-secure households relative to the insecure households. The positive effect of education on the reduction of food insecurity has been indicated by previous researchers (18, 25, 27, 59, 62–66). Except in food insecure households, the effect of employment on households’ food security is significant in vulnerable and secure households, and its effect increases as quantiles of food security increase. This result is also supported by previous researchers (27, 58, 65, 67).

Household size has a significant positive impact across all levels of the food security score distribution, but the magnitude of this effect decreases in food secure households. This result is in contrast with previous studies (63, 68, 69). This may be attributed to children being seen as a source of wealth in Ethiopia, and they work on farms or in any business area to bring money to the family. The effect of the sex of the household head is not significant even though male-headed households have a better food security score as suggested by many studies (64, 65, 70). The age of the household head has a higher negative significant effect on lower quantiles of food security score. The effect of age is indicated by previous studies (27, 64, 65).

The sanitation and drinking water component scores have a significantly higher effect in food-secure households, whereas the effect decreases in insecure households. Previous studies also point out drinking water and sanitation as an input in the reduction of food insecurity studies (18, 22, 71). Facing health problems has a high effect on the vulnerable to food insecure households relative to the food insecure and secure households. The health problem effect on food insecurity was also reported by previous researchers (20, 25).

The shock that occurred in the household is significantly higher in food-insecure households, and its effect decreases in food-secure households. The effect of shock, like a rise in the price of food items, an increase in the price of inputs, illness of a household member, and drought, on food insecurity was also indicated by several researchers (22, 70–72). The effect of the dependency ratio is significantly high on food-insecure households, whereas its effect decreases as the level of food security increases. The negative effect of the dependency ratio on the reduction of food insecurity is indicated by a previous study (22, 27). Adult equivalence has a negative effect all over the levels of the food security score distribution. The higher adult equivalence or a large number of consumption units per household reduced food security score over all levels and previous studies also indicated the negative effect of higher adult equivalence on food insecurity reduction (25, 73).

Farming-type livestock or livestock with cropping has a significantly higher effect on higher quintiles of food security scores, whereas the effect decreases on the lower quantiles. Many studies agreed on the adoption of drought-resistant farming in food-insecure areas (58, 62, 64, 69, 74). Having a small-size land either owned or rented for cropping has a higher negative effect on insecure and vulnerable households, and the effect decreases and becomes insignificant in secure households/upper quantiles. This result is supported by Cheema et al. (2020) (27, 58, 66, 69, 71, 74), and agrees with the Ethiopian government’s initiative on integrated farming.

The agricultural package or component score of advisory service, extension program, credit for agriculture, and crop rotation has a significantly higher effect on vulnerable and secure households. The improvement obtained in food security from agricultural package implementation is also indicated by previous researchers (58, 64). The usage of fertilizers has a significantly high effect on food-secure households, and its effect decreases in food-insecure households. This result suggested that cropping using fertilizers can lead to a higher level of food security score. Previous studies also supported the importance of fertilizer for food insecurity reduction (66, 69, 75).

Soil property component score has a significant positive effect on food-insecure and vulnerable households; this implies that soil quality has a positive effect on the reduction of food insecurity. This result is aligned with previous studies (58, 64) and the Ethiopian government’s agricultural package policies on soil conservation by planting trees, grass, and terrace farming. The component score for agroecological and distance from border-related and the component score for rainfall and greens have a significantly higher effect on the food secure households and its effect decreases on the food insecure households. This result is supported by previous studies (60, 64, 68, 70). The non-agricultural business and related factors component scores have a significant positive effect on vulnerable households and its effect declined to the end of the quantiles of the food security score. This result is aligned with previous studies (27, 63, 64). The component score of irrigation, mixed cropping, crop damage, and erosion has a significant effect on lower quantiles (25th and 35th) of the food security score. This result is supported by previous studies (22, 60, 63, 76, 77).

Information sources, housing quality, and the electronic and furniture-related component scores have an increasingly significant positive impact across the quantiles of the food security score distribution. This result is aligned with previous studies (18, 22, 25, 65). The severity of the coping strategy has a significant effect on vulnerable households. Previous research found that different coping strategies are applied by households based on the magnitude of food shortage (22, 78).

The corrected WFP FCS cut points split out the significant association of ability to read and write with vulnerable and food-secure households, residence in urban areas with food security, fertilizer usage with food-secure households, farming livestock or/and crop with food-secure and vulnerable households, shock with food insecure households, dependency ratio with food insecure and vulnerable households, sanitation-related with food insecure and vulnerable households, age of household head with food insecure households, and health problem with vulnerable households.

Compared to the WFP cut points for FCS, the effect-driven approach cut points used in this study bring a wider interval for food insecure households, with the same interval range for vulnerable households. The result revealed that measuring food insecurity in Ethiopia by FCS with WFP cut points (28 and 42) underestimates households’ food insecurity levels by a score of seven. If we adopt the WFP FCS cut-points 28 and 42; some households will wrongly assigned on each levels, especially the proportion of secured households will inflate and the proportion of food insecure households will be under estimated, and factors will wrongly recommend for their influence on the levels of food security score. For example, if we use the WFP cut points, the health problem of the household head will have no totally significant effect on insecure households, small-size land ownership and the dependency ratio will also be recommended as an influential factor on food secure households, and residence in urban areas have no statically significant difference on being vulnerable to food insecurity relative to rural households. Previous studies also suggested further work on the need for threshold correction due to local factors such as cultural diversity, which resulted in consuming small amounts of food. Similarly, WFP adjusted the FCS by a score of seven for a high frequency of consumption of small amounts of sugar and oil and gave the alternate cut-offs to be 28 and 42 (29). Previous research also reported households’ FCS cut points to be 45 and 61 for Jordan (31) and 32 and 43 for Laos due to cultural disparities (30).

## Strengths, limitations, and future work

Previous research on food security used cross-sectional data and focused on investigating the effects of factors on the lower quantile of food security (food-insecure only). This paper has several strengths; even if the currently available data have smaller replication, it is the only long term available panel data. Furthermore, this paper addressed measurement problems based on effect-driven classification of quantiles, and identifies mitigations at a local level by considering the evolutional variability (sustainability over time) of food security score over the quantiles. As a result, this paper found the major factors and a universally accepted local threshold corrected to local factors for food insecure, vulnerable, and secure households by considering the longitudinal effect that can be an input for future researchers and policymakers.

We have used the available data that is older than 7 years since recent data is not yet collected by the concerned body due to many problems faced in Ethiopia including political instability, war, and displacement. On the other hand, even if the sample size is large enough (*n* = 3,835), due to the small number of repeated measurements in households (in 2012, 2014, and 2016), the likelihood approach error covariance model does not converge, and error covariance modeling is done by free the distribution covariance structure.

Therefore, as future work one can extend the work using sufficiently repeated measurements based on the panel data that will be released in the future. There is also a need for comprehensive research that considers cultural disparities across nations which affect consumption patterns to fix a universal threshold or some robust estimate.

## Conclusion

The effect-driven approach cut points for FCS leveled the food security of Ethiopian households into three with cut points 35.5 and 49, which brings wider intervals for food insecure households and the same interval size for vulnerable households, while the WFP cut points (28 and 42) underestimates households’ food insecurity levels by a score of seven. Therefore, applying WFP cut points will wrongly assign households on each level; especially, the proportion of secure households will inflate and the proportion of food insecure households will be underestimated, and factors will be wrongly recommended for their influence on levels of food security score.

Ethiopian households’ food security showed improvement over time across all quintiles of food security score distribution. The progress is higher in food insecure and secure compared to vulnerable households; mainly the improvement in the food secured households is higher. Food security scores differ across regions throughout the quantiles of the FCS. Households living in urban areas have better food security compared to rural areas.

In general, the quantile regression approach adjusts the WFP-FCS by adjusting for local situations/factors. Accordingly, this study has agreed with the suggestion of previous studies and suggested the need for a universally accepted threshold corrected for local factors, like cultural disparity which resulted in different consumption patterns in the standardization of food security score.

The proposed approach is constrained by driving factors in leveling food security and identifies mitigation at a local level to eliminate food insecurity for better public health. The suggested result of this study can help policymakers to intervene in mitigation at each level for the improvement of households’ food security levels for better public health and social security in Ethiopia. Especially, integrated farming “ኩታ ገጠም እርሻ” using irrigation, mitigation for controlling shocks, and reducing dependency ratio can save food insecure households from severe risks. Correspondingly, to achieve better households’ food security, working on access to education, urbanization, sanitation and drinking water, infrastructure, fertilizer delivery and farming of both livestock and crops, protection of the environment, and land degradation is essential.

## Data availability statement

The original contributions presented in the study are included in the article/Supplementary material, further inquiries can be directed to the corresponding author.

## Author contributions

HW was involved in the conception, data management, data analysis, and interpretation of this study. TZ contributed to the conception, design, review, and revisions of the manuscript. AM contributed to the interpretation of the analysis and review of the manuscript. ZD contributed to the data management, interpretation, and revisions of the manuscript. All authors have read and approved the final manuscript.

## Conflict of interest

The authors declare that the research was conducted in the absence of any commercial or financial relationships that could be construed as a potential conflict of interest.

## Publisher’s note

All claims expressed in this article are solely those of the authors and do not necessarily represent those of their affiliated organizations, or those of the publisher, the editors and the reviewers. Any product that may be evaluated in this article, or claim that may be made by its manufacturer, is not guaranteed or endorsed by the publisher.

## Footnotes

^1^ https://stage-data.kimetrica.com/id/dataset

## Abbreviations

AGSS: Annual agricultural sample survey
AIC: Akaike information criterion
CSA: Central statistical agency
EAs: enumeration areas
FAO: Food and agricultural organization
FCS: Food consumption score
FCSL: Food insecurity score levels
ICC: intra correlation coefficient
LMM: Linear mixed linear model
*lqmm*: Linear quantile mixed model
HH: Household
PCA: Principal component analysis
PPS: probability proportional to the size of the population
SNNP: southern nations and nationalities people of Ethiopia
WFP: World food program
