# Supplementary material for: Household food insecurity levels in Ethiopia: quantile regression approach
Source: Front Public Health. 2023 Jul 10;11:1173360. doi: 10.3389/fpubh.2023.1173360 (PMC10365274; doi:10.3389/fpubh.2023.1173360)
Supplement: Supplementary file 1 [file Data_Sheet_1.docx]

# Appendix

## Appendix Table

Appendix Table 1: The list of all 42 variable (1^st^ column) & CI for Linear mixed model (LMM) coefficient (2^nd^ column), and CI for longitudinal quantile regression model coefficients estimates (3^rd^ to 8^th^ columns).

| Estimate | LMM | q0.05 | q0.1 | q0.25 | q0.35 | q0.5 | q0.75 | q0.9 | q0.95 |
| --- | --- | --- | --- | --- | --- | --- | --- | --- | --- |
| (Intercept) | -1434.22(-1992.40― -876.03) | -1437.30(-2116.80 ― -757.85) | -1421.90(-1971.40 ― -872.32) | -1432.50(-2213.70 ― -651.38) | -1438.50(-2128.10 ― -748.93) | -1439.10(-2224.80 ― -653.27) | -1440.00(-2163.20 ― -716.86) | -1425.60(-2144.30 ― -706.83) | -1437.20(-2167.40 ― -707.05) |
| Year (x_1_) | 0.74(0.46―1.01) | 0.72(0.39 ― 1.06) | 0.72(0.45 ― 0.99) | 0.74(0.35 ― 1.12) | 0.74(0.39 ― 1.08) | 0.74(0.35 ― 1.13) | 0.74(0.38 ― 1.09) | 0.74(0.38 ― 1.09) | 0.74(0.38 ― 1.10) |
| Mixed Cropping (x_2_) | 1176.34(402.51 ― 1950.23) | 1258.10(521.62 ― 1994.67) | 1259.70(434.12 ― 2085.33) | 1259.20(507.37 ― 2011.11) | 1258.20(566.08 ― 1950.39) | 1258.50(523.45 ― 1993.60) | 1259.80(393.93 ― 2125.66) | 1260.20(400.58 ― 2119.73) | 1259.50(383.38 ― 2135.71) |
| Urban Vs Rural (x_3_) | 0.72(-0.78 ― 2.21) | 7.89(2.08 ― 13.70) | 4.23(-1.46 ― 9.91) | 0.85(-2.32 ― 4.01) | 0.27(-1.65 ― 2.19) | 2.06(-0.41 ― 4.54) | 4.09(1.79 ― 6.39) | 4.60(1.07 ― 8.12) | 12.44(7.33 ― 17.55) |
| Sex of household head (x_4_) | 0.20(-0.78 ― 1.18) | 0.22(-3.22 ― 3.66) | 0.49(-2.10 ― 3.09) | -0.08(-2.04 ― 1.88) | 0.65(-0.85 ― 2.14) | -0.61(-2.01 ― 0.80) | 0.50(-1.12 ― 2.13) | 0.98(-1.30 ― 3.26) | 2.21(-1.15 ― 5.57) |
| Read & Write (x_5_) | 1.95(1.10 ― 2.80) | 4.31(1.50 ― 7.12) | 0.65(-2.22 ― 3.52) | 0.55(-1.26 ― 2.35) | -1.98(-3.96 ― 0.00) | 1.98(0.80 ― 3.17) | 2.59(0.94 ― 4.24) | 3.41(1.24 ― 5.57) | 7.31(4.33 ― 10.30) |
| Shock (x_6_) | -1.08(-1.74 ― -0.41) | -3.04(-5.22 ― -0.85) | -3.33(-5.67 ― -0.98) | -1.43(-3.06 ― 0.20) | -0.58(-1.81 ― 0.64) | -1.89(-2.89 ― -0.90) | -0.98(-2.14 ― 0.17) | -0.66(-1.94 ― 0.61) | -0.16(-2.40 ― 2.07) |
| Fertilizer (x_7_) | 0.73(-0.27 ― 1.73) | 1.70(-1.14 ― 4.55) | -0.65(-4.32 ― 3.02) | -0.18(-2.25 ― 1.89) | 1.44(0.09 ― 2.79) | 0.20(-1.19 ― 1.58) | 2.28(0.86 ― 3.70) | 2.24(0.08 ― 4.40) | 5.42(2.41 ― 8.43) |
| Adult equivalence (x_8_) | -1.14(-2.08 ― -0.19) | -1.65(-3.18 ― -0.13) | -1.45(-3.23 ― 0.34) | -1.48(-2.82 ― -0.14) | -0.80(-2.15 ― 0.54) | -1.31(-2.47 ― -0.15) | -0.99(-2.11 ― 0.13) | -1.07(-2.03 ― -0.11) | -1.74(-3.19 ― -0.29) |
| Age of household head (x_9_) | -0.01(-0.04 ― 0.02) | -0.19(-0.31 ― -0.06) | -0.08(-0.17 ― 0.01) | -0.07(-0.12 ― -0.03) | -0.03(-0.07 ― 0.00) | -0.01(-0.04 ― 0.02) | -0.02(-0.06 ― 0.03) | 0.00(-0.06 ― 0.06) | 0.02(-0.06 ― 0.09) |
| Assistance indicator (x_10_) | 0.32(-0.64 ― 1.28) | 3.10(0.28 ― 5.91) | 4.76(0.65 ― 8.88) | 0.00(-2.66 ― 2.66) | 0.35(-1.21 ― 1.91) | 0.12(-1.34 ― 1.59) | 1.30(-0.54 ― 3.13) | 2.52(-0.64 ― 5.68) | -0.79(-3.90 ― 2.32) |
| Copping Strategy Index (x_11_) | -0.03(-0.06 ― -0.01) | -0.03(-0.12 ― 0.06) | -0.12(-0.36 ― 0.12) | -0.14(-0.26 ― -0.03) | -0.12(-0.20 ― -0.05) | -0.06(-0.11 ― -0.02) | -0.05(-0.11 ― 0.01) | -0.05(-0.12 ― 0.01) | 0.04(-0.09 ― 0.17) |
| Dependency Ratio (x_12_) | -0.30(-0.58 ― -0.02) | -0.30(-1.27 ― 0.68) | -1.22(-2.26 ― -0.17) | -1.05(-1.72 ― -0.38) | -0.71(-1.24 ― -0.18) | -0.45(-0.90 ― 0.00) | -0.10(-0.59 ― 0.39) | -0.16(-0.89 ― 0.57) | 0.89(-0.28 ― 2.07) |
| Eat the same diet (x_13_) | -1.26(-2.99 ― 0.48) | -0.64(-6.74 ― 5.46) | -7.58(-16.78 ― 1.61) | -2.22(-6.11 ― 1.66) | -1.15(-4.21 ― 1.90) | -2.00(-3.70 ― -0.30) | 1.14(-1.49 ― 3.76) | -3.80(-6.52 ― -1.07) | -1.28(-5.91 ― 3.36) |
| Employed (x_14_) | 2.34(1.05 ― 3.63) | -2.75(-7.19 ― 1.68) | 3.38(-1.43 ― 8.19) | 3.14(0.76 ― 5.52) | 3.21(1.37 ― 5.05) | 3.91(1.87 ― 5.94) | 4.65(2.37 ― 6.93) | 5.35(1.75 ― 8.96) | -0.62(-6.24 ― 5.01) |
| Farm Type (x_15_):[Livestock] | 2.63(0.67 ― 4.60) | 15.84(7.58 ― 24.09) | 4.65(-1.13 ― 10.42) | 5.90(1.17 ― 10.64) | 6.44(3.19 ― 9.68) | 8.82(5.07 ― 12.56) | 8.87(5.93 ― 11.82) | 6.60(0.22 ― 12.97) | 13.32(6.27 ― 20.37) |
| Farm Type (x_15_) :[Both farms] | 1.31(0.10 ― 2.52) | 2.74(-1.71 ― 7.19) | -0.79(-4.60 ― 3.01) | 1.33(-1.30 ― 3.96) | 1.07(-0.54 ― 2.69) | 1.77(0.51 ― 3.04) | 4.24(2.61 ― 5.87) | 2.88(-0.05 ― 5.81) | 1.89(-0.96 ― 4.74) |
| Food worry (x_16_) | -2.70(-3.72 ― -1.68) | -0.59(-3.47 ― 2.28) | 2.74(-1.58 ― 7.06) | -1.43(-4.09 ― 1.23) | -2.02(-3.94 ― -0.10) | -0.96(-2.54 ― 0.62) | -0.40(-2.30 ― 1.49) | -0.69(-3.17 ― 1.80) | 0.04(-3.44 ― 3.51) |
| Get Health Assistance (x_17_) | 1.83(0.86 ― 2.80) | 2.95(0.25 ― 5.66) | 0.50(-2.13 ― 3.12) | 1.84(-0.03 ― 3.71) | 1.20(-0.59 ― 3.00) | 1.03(-0.21 ― 2.27) | 1.81(0.29 ― 3.33) | 2.43(-0.11 ― 4.98) | 5.07(1.34 ― 8.79) |
| Health problem (x_18_) | -1.36(-2.33 ― -0.39) | 1.80(-1.08 ― 4.68) | -0.63(-3.61 ― 2.35) | -1.79(-3.64 ― 0.06) | -2.19(-4.17 ― -0.20) | -1.81(-3.15 ― -0.46) | -0.64(-2.21 ― 0.92) | -0.55(-2.81 ― 1.70) | 1.86(-1.36 ― 5.08) |
| Household size (x_19_) | 1.51(0.73 ― 2.29) | 1.95(0.63 ― 3.26) | 1.78(0.41 ― 3.14) | 1.39(0.37 ― 2.40) | 1.04(-0.02 ― 2.11) | 1.50(0.60 ― 2.39) | 1.29(0.32 ― 2.26) | 1.60(0.69 ― 2.52) | 1.23(0.09 ― 2.37) |
| Small size land ownership (x_20_) | -2.36(-3.58 ― -1.14) | -4.20(-7.44 ― -0.96) | -3.86(-7.98 ― 0.27) | -3.78(-6.60 ― -0.97) | -2.74(-4.81 ― -0.67) | -2.61(-3.97 ― -1.25) | -0.96(-3.00 ― 1.09) | -2.17(-4.83 ― 0.49) | -2.18(-5.72 ― 1.35) |
| Number of Livestock (x_21_) | 0.05(0.02 ― 0.08) | 0.05(-0.05 ― 0.15) | 0.08(-0.15 ― 0.31) | 0.06(-0.11 ― 0.22) | 0.02(-0.06 ― 0.09) | 0.06(0.00 ― 0.12) | 0.08(0.02 ― 0.15) | 0.06(-0.03 ― 0.15) | 0.01(-0.14 ― 0.15) |
| Meals eaten by Childe (x_22_) | -0.13(-0.43 ― 0.18) | 0.28(-0.66 ― 1.23) | 0.03(-1.11 ― 1.16) | -0.82(-1.41 ― -0.23) | -0.44(-1.14 ― 0.25) | -0.25(-0.60 ― 0.11) | 0.13(-0.42 ― 0.67) | -0.18(-0.79 ― 0.43) | -0.30(-1.30 ― 0.70) |
| Meals eaten by young (x_23_) | 1.04(0.39 ― 1.69) | 1.39(-0.66 ― 3.44) | 0.69(-1.68 ― 3.06) | 0.66(-0.63 ― 1.95) | 0.88(-0.20 ― 1.96) | 1.22(0.45 ― 2.00) | 1.07(0.24 ― 1.90) | 1.69(0.42 ― 2.96) | 1.10(-1.30 ― 3.49) |
| Soil property related (x_24_) | 0.62(0.16 ― 1.07) | 2.39(0.70 ― 4.09) | 0.75(-0.57 ― 2.06) | 0.85(-0.04 ― 1.75) | 0.64(-0.07 ― 1.36) | 0.40(-0.16 ― 0.97) | 1.44(0.32 ― 2.57) | 1.49(0.09 ― 2.89) | 1.55(-0.84 ― 3.95) |
| Agro-ecological & distance from border related (x_25_) | -1.11(-1.70 ― -0.53) | 0.78(-1.02 ― 2.58) | 0.25(-1.80 ― 2.29) | 0.71(-0.47 ― 1.88) | 0.24(-0.71 ― 1.19) | -0.91(-1.58 ― -0.24) | -0.95(-1.86 ― -0.04) | -0.52(-2.04 ― 1.00) | -2.68(-4.17 ― -1.20) |
| Rainfall & greens related (x_26_) | -1.00(-1.61 ― -0.40) | -0.75(-3.05 ― 1.54) | -0.71(-2.40 ― 0.99) | -1.40(-2.46 ― -0.33) | -0.86(-1.52 ― -0.20) | -1.15(-1.92 ― -0.38) | -1.18(-2.11 ― -0.25) | -1.77(-3.40 ― -0.14) | -1.30(-2.76 ― 0.17) |
| Rooting conditions & workability related (x_27_) | 0.11(-0.35 ― 0.57) | -1.42(-2.90 ― 0.07) | 0.15(-1.43 ― 1.73) | -0.19(-1.23 ― 0.84) | 0.20(-0.52 ― 0.91) | 0.03(-0.61 ― 0.67) | -0.31(-1.14 ― 0.52) | -0.48(-1.62 ― 0.65) | -0.24(-1.99 ― 1.51) |
| Distance from market, road & population center related (x_28_) | -0.16(-0.60 ― 0.28) | 1.83(0.16 ― 3.49) | 0.52(-0.72 ― 1.75) | 0.49(-0.38 ― 1.36) | 0.52(-0.21 ― 1.25) | -0.41(-1.02 ― 0.20) | -0.11(-0.82 ― 0.59) | -0.03(-1.15 ― 1.08) | -0.70(-2.86 ― 1.46) |
| Terrain Roughness & wetness related (x_29_) | 0.51(0.09 ― 0.93) | 0.52(-0.76 ― 1.80) | 0.50(-0.70 ― 1.69) | 0.18(-0.56 ― 0.91) | 0.42(-0.25 ― 1.09) | 0.42(-0.18 ― 1.02) | 0.47(-0.29 ― 1.23) | 0.56(-0.39 ― 1.52) | -0.11(-1.32 ― 1.10) |
| Agricultural package related (x_30_) | 0.23(-0.27 ― 0.73) | 0.99(-0.63 ― 2.60) | 1.14(-0.71 ― 2.98) | 1.41(0.65 ― 2.17) | 0.44(-0.37 ― 1.25) | 1.08(0.38 ― 1.78) | 0.70(-0.04 ― 1.44) | 0.65(-0.09 ― 1.38) | 0.70(-0.87 ― 2.27) |
| Agricultural material related (x_31_) | 0.51(0.09 ― 0.93) | 0.31(-1.33 ― 1.95) | 0.33(-0.88 ― 1.53) | 0.97(0.01 ― 1.92) | 0.14(-0.38 ― 0.67) | 0.49(-0.05 ― 1.02) | 0.05(-0.67 ― 0.76) | 0.54(-0.30 ― 1.38) | 0.99(-0.16 ― 2.14) |
| Blanket & Bed related (x_32_) | 1.55(1.20 ― 1.91) | 1.65(0.45 ― 2.85) | 1.83(0.63 ― 3.04) | 1.80(0.98 ― 2.62) | 1.55(0.96 ― 2.15) | 1.61(1.05 ― 2.18) | 1.87(1.30 ― 2.44) | 1.79(0.88 ― 2.70) | 2.56(1.29 ― 3.82) |
| Drinking Water (x_33_) | 0.57(0.19 ― 0.95) | -0.04(-1.35 ― 1.28) | -0.83(-2.13 ― 0.46) | -0.09(-0.94 ― 0.76) | 0.00(-0.56 ― 0.56) | 0.41(-0.20 ― 1.01) | 0.15(-0.45 ― 0.74) | 0.47(-0.41 ― 1.35) | 1.28(0.14 ― 2.43) |
| Electronic & Furniture related (x_34_) | 2.22(1.83 ― 2.61) | 2.41(1.14 ― 3.68) | 1.15(-0.01 ― 2.30) | 1.70(0.90 ― 2.50) | 2.36(1.73 ― 2.98) | 2.00(1.41 ― 2.58) | 2.79(1.82 ― 3.77) | 3.14(2.17 ― 4.11) | 5.78(4.13 ― 7.43) |
| Housing Quality related (x_35_) | 3.61(3.21 ― 4.02) | 2.56(1.21 ― 3.91) | 2.24(0.90 ― 3.58) | 3.31(2.31 ― 4.31) | 3.51(2.87 ― 4.15) | 3.26(2.72 ― 3.80) | 3.25(2.54 ― 3.95) | 3.55(2.34 ― 4.77) | 3.45(2.45 ― 4.45) |
| Information Source related (x_36_) | 3.26(2.89 ― 3.64) | 1.96(0.99 ― 2.93) | 2.21(0.96 ― 3.46) | 2.25(1.55 ― 2.95) | 3.08(2.29 ― 3.86) | 3.13(2.66 ― 3.59) | 3.42(2.67 ― 4.17) | 4.20(3.43 ― 4.97) | 3.27(2.19 ― 4.35) |
| Irrigation and related (x_37_) | -0.18(-0.75 ― 0.38) | -0.02(-2.32 ― 2.29) | 0.74(-1.09 ― 2.56) | 1.67(0.66 ― 2.67) | 0.01(-0.79 ― 0.82) | -0.15(-0.97 ― 0.67) | -0.15(-0.95 ― 0.65) | -0.48(-1.38 ― 0.43) | 0.66(-1.33 ― 2.66) |
| Non-agricultural Business related (x_38_) | 1.07(0.70 ― 1.43) | -1.04(-2.35 ― 0.26) | 0.54(-0.57 ― 1.65) | 0.67(-0.16 ― 1.50) | 0.78(0.34 ― 1.23) | 0.90(0.39 ― 1.41) | 0.84(0.12 ― 1.55) | 0.47(-0.40 ― 1.34) | 0.26(-0.90 ― 1.42) |
| Productive Asset related (x_39_) | -0.11(-0.44 ― 0.22) | 0.38(-0.48 ― 1.24) | -0.20(-1.33 ― 0.94) | -0.32(-1.20 ― 0.56) | -0.51(-1.06 ― 0.05) | -0.09(-0.74 ― 0.55) | -0.06(-0.94 ― 0.83) | 0.25(-0.91 ― 1.42) | 1.23(-0.25 ― 2.72) |
| PSNP (x_40_) | -0.22(-0.60 ― 0.15) | -0.71(-1.98 ― 0.55) | -0.16(-1.35 ― 1.02) | -0.70(-1.46 ― 0.06) | -0.57(-1.26 ― 0.13) | -0.18(-0.70 ― 0.33) | -0.06(-0.94 ― 0.81) | -0.74(-1.50 ― 0.02) | 0.41(-0.90 ― 1.72) |
| Sanitation related (x_41_) | 1.32(0.95 ― 1.70) | 0.87(-0.36 ― 2.09) | 0.57(-0.51 ― 1.64) | 0.72(-0.11 ― 1.56) | 1.19(0.45 ― 1.92) | 1.32(0.83 ― 1.81) | 1.43(0.75 ― 2.11) | 1.55(0.78 ― 2.31) | 3.29(1.75 ― 4.83) |
| Region (x_42_): [Amhara ] | -2.24(-5.44 ― 0.96) | 8.02(-3.68 ― 19.72) | 7.75(0.68 ― 14.82) | -1.42(-5.12 ― 2.27) | -1.88(-5.38 ― 1.63) | -0.03(-4.11 ― 4.05) | 2.86(-0.71 ― 6.43) | 1.17(-5.55 ― 7.89) | 6.84(0.78 ― 12.89) |
| [B.Gumuz] | -3.40(-7.24 ― 0.45) | 9.96(-2.34 ― 22.26) | 12.53(3.08 ― 21.98) | 10.38(4.83 ― 15.93) | 6.15(1.39 ― 10.91) | 4.67(-0.24 ― 9.57) | 5.37(-2.18 ― 12.91) | 9.20(-3.34 ― 21.74) | 16.44(5.02 ― 27.86) |
| [Diredwa] | 4.74(1.19 ― 8.29) | 24.39(13.21 ― 35.56) | 23.43(14.98 ― 31.89) | 12.98(4.99 ― 20.97) | 15.19(10.13 ― 20.26) | 12.89(7.40 ― 18.39) | 16.27(6.82 ― 25.72) | 22.75(9.26 ― 36.23) | 26.72(14.66 ― 38.78) |
| [Gambelia] | 0.17(-3.66 ― 3.99) | 16.51(0.90 ― 32.11) | 18.52(6.94 ― 30.10) | 15.19(7.78 ― 22.60) | 12.07(6.90 ― 17.24) | 8.51(3.48 ― 13.55) | 11.94(2.61 ― 21.26) | 13.92(0.14 ― 27.69) | 22.55(9.69 ― 35.42) |
| [Harari] | 1.94(-1.73 ― 5.61) | 16.03(2.07 ― 29.98) | 20.08(10.21 ― 29.96) | 9.70(2.79 ― 16.61) | 11.25(5.70 ― 16.79) | 9.61(3.58 ― 15.63) | 15.40(5.51 ― 25.29) | 19.82(6.46 ― 33.18) | 25.90(12.13 ― 39.66) |
| [Oromia] | 4.07(0.80 ― 7.34) | 10.67(-2.52 ― 23.86) | 15.04(8.84 ― 21.24) | 5.08(1.01 ― 9.14) | 4.75(1.04 ― 8.45) | 6.58(2.59 ― 10.56) | 9.68(5.61 ― 13.75) | 9.99(4.44 ― 15.53) | 15.75(9.93 ― 21.57) |
| [SNNP] | -6.31(-9.59 ― -3.03) | 1.04(-7.82 ― 9.91) | 4.68(-2.55 ― 11.92) | -6.84(-10.78 ― -2.90) | -6.17(-10.10 ― -2.24) | -4.37(-8.70 ― -0.05) | -1.44(-5.30 ― 2.43) | -0.88(-6.27 ― 4.51) | 3.51(-1.41 ― 8.43) |
| [Somalie] | 3.86(1.07 ― 6.64) | 16.96(7.58 ― 26.33) | 13.80(7.49 ― 20.11) | 7.86(2.25 ― 13.47) | 8.16(3.17 ― 13.15) | 8.09(4.20 ― 11.98) | 10.98(5.67 ― 16.28) | 7.96(-1.16 ― 17.08) | 19.82(11.51 ― 28.13) |
| [Tigray] | -4.41(-7.50 ― -1.32) | 4.76(-4.09 ― 13.61) | 8.86(0.34 ― 17.39) | -1.55(-5.98 ― 2.89) | -2.84(-6.22 ― 0.54) | -2.37(-6.75 ― 2.01) | 3.14(-0.99 ― 7.26) | 5.51(-1.91 ― 12.93) | 13.37(7.26 ― 19.47) |
| year:Mixed Cropping | -0.58(-0.97 ― -0.20) | -0.62(-0.99 ― -0.26) | -0.62(-1.04 ― -0.21) | -0.63(-1.00 ― -0.25) | -0.63(-0.97 ― -0.28) | -0.62(-0.99 ― -0.26) | -0.62(-1.05 ― -0.19) | -0.63(-1.05 ― -0.20) | -0.62(-1.06 ― -0.19) |

Appendix Table 2: Confidence Interval for linear quantile regression coefficients estimate.

| Estimate | q0.05 | q0.1 | q0.25 | q0.35 | q0.5 | q0.75 | q0.9 | q0.95 |
| --- | --- | --- | --- | --- | --- | --- | --- | --- |
| (Intercept) | -1042.00(-1893.00 ― -164.80) | -1256.00(-1963.00 ― -270.80) | -1556.00(-2177.00 ― -779.4) | -1327.00(-1825.00 ― -836.30) | -1384.00(-2014.00 ― -875.90) | -1322.00(-2278.00 ― -748.10) | -773.50(-2124.00 ― 224.60) | -1371.00(-2495.00 ― 295.70) |
| Year (x_1_) | 0.53(0.09 ― 0.95) | 0.64(0.15 ― 0.99) | 0.79(0.40 ― 1.092 | 0.68(0.43 ― 0.92) | 0.71(0.46 ― 1.02) | 0.68(0.39 ― 1.16) | 0.41(-0.08 ― 1.08) | 0.71(-0.11 ― 1.27) |
| Mixed Cropping (x_2_) | 586.40(-666.90 ― 1675.00) | 497.70(-435.60 ― 1944.00) | 1436.00(434.50 ― 2266.00) | 1014.00(215.70 ― 1610.00) | 852.30(29.92 ― 1906.00) | 1621.00(572.50 ― 2682.00) | 720.70(-747.70 ― 2317.00) | 1424.00(-300.80 ― 3360.00) |
| Urban Vs Rural (x_3_) | -0.88(-2.60 ― 0.96) | 0.97(-0.86 ― 2.51) | -0.10(-1.85 ― 1.44) | -0.53(-1.93 ― 0.39) | -0.79(-2.43 ― 0.45) | 0.22(-1.32 ― 1.79) | 2.29(0.04 ― 5.15) | 2.49(-0.81 ― 4.63) |
| Sex of household head (x_4_) | 0.29(-1.06 ― 1.04) | -0.53(-1.61 ― 0.33) | 0.22(-0.59 ― 1.00) | 0.26(-0.45 ― 0.98) | -0.10(-1.12 ― 0.68) | 0.31(-0.42 ― 1.22) | -0.01(-1.83 ― 1.75) | 1.03(-0.79 ― 2.93) |
| Read & Write (x_5_) | 2.06(1.07 ― 3.44) | 1.38(0.35 ― 2.54) | 1.54(0.85 ― 2.46) | 1.55(0.82 ― 2.13) | 2.18(1.23 ― 2.90) | 3.02(2.14 ― 4.20) | 2.95(1.33 ― 4.32) | 4.15(2.38 ― 5.68) |
| Shock (x_6_) | 0.16(-0.69 ― 1.10) | 0.24(-0.75 ― 1.35) | -0.23(-0.96 ― 0.48) | -0.19(-0.83 ― 0.42) | -0.83(-1.55 ― -0.23) | -1.11(-1.94 ― -0.42) | -1.16(-2.75 ― -0.22) | -2.06(-3.56 ― -0.41) |
| Fertilizer (x_7_) | -0.87(-2.26 ― 0.15) | -0.75(-2.09 ― 0.55) | 0.41(-0.78 ― 1.48) | 0.49(-0.32 ― 1.24) | 0.34(-0.57 ― 1.33) | 2.04(1.21 ― 3.24) | 1.77(0.32 ― 3.47) | 1.37(-0.45 ― 3.60) |
| Adult equivalence (x_8_) | -0.59(-1.51 ― 0.55) | -1.17(-2.05 ― 0.19) | -0.67(-1.80 ― 0.20) | -0.92(-1.58 ― -0.25) | -0.67(-1.66 ― 0.33) | -1.60(-2.35 ― -0.36) | -1.43(-2.80 ― -0.03) | -1.56(-3.53 ― 0.24) |
| Age of household head (x_9_) | 0.00(-0.03 ― 0.03) | 0.00(-0.04 ― 0.03) | -0.01(-0.03 ― 0.02) | -0.01(-0.02 ― 0.01) | -0.02(-0.04 ― 0.01) | 0.01(-0.01 ― 0.04) | -0.02(-0.05 ― 0.03) | -0.02(-0.07 ― 0.04) |
| Assistance indicator (x_10_) | 1.51(-0.13 ― 2.93) | 0.92(-0.34 ― 2.20) | -0.20(-1.28 ― 0.60) | -0.36(-1.14 ― 0.43) | -0.18(-1.21 ― 0.62) | 0.10(-1.13 ― 1.51) | -0.11(-1.53 ― 1.81) | 0.04(-2.54 ― 1.78) |
| Copping Strategy Index (x_11_) | -0.04(-0.08 ― 0.00) | -0.02(-0.06 ― 0.01) | -0.04(-0.07 ― -0.01) | -0.04(-0.07 ― -0.01) | -0.03(-0.07 ― 0.00) | -0.04(-0.08 ― 0.00) | -0.06(-0.11 ― -0.01) | -0.07(-0.12 ― 0.01) |
| Dependency Ratio (x_12_) | -0.31(-0.73 ― -0.03) | -0.36(-0.68 ― -0.08) | -0.34(-0.76 ― 0.03) | -0.33(-0.62 ― 0.03) | -0.07(-0.43 ― 0.13) | -0.04(-0.37 ― 0.30) | 0.00(-0.48 ― 0.39) | -0.26(-0.77 ― 0.08) |
| Eat the same diet (x_13_) | -1.36(-3.67 ― 1.65) | -1.34(-4.07 ― 0.54) | -1.99(-4.06 ― -0.06) | -1.94(-3.28 ― -0.27) | -1.29(-2.90 ― 1.10) | -1.40(-5.16 ― 1.18) | -1.30(-4.46 ― 0.83) | 1.59(-3.46 ― 3.31) |
| Employed (x_14_) | 0.78(-0.99 ― 2.67) | 1.97(-0.28 ― 3.43) | 0.84(-0.57 ― 2.42) | 1.78(0.97 ― 3.17) | 2.38(0.97 ― 3.74) | 2.83(1.31 ― 4.17) | 0.77(-1.16 ― 3.59) | -0.31(-2.30 ― 3.06) |
| Farm Type (x_15_):[Livestock] | -1.51(-4.12 ― 0.51) | -0.84(-3.03 ― 2.47) | 2.17(0.12 ― 4.62) | 2.36(0.77 ― 4.08) | 3.87(2.22 ― 5.89) | 4.87(3.03 ― 6.69) | 4.10(1.12 ― 9.12) | 5.74(0.82 ― 8.93) |
| Farm Type (x_15_) :[Both farms] | -0.29(-2.07 ― 1.14) | 0.32(-0.91 ― 1.86) | 1.42(0.13 ― 2.73) | 1.61(0.77 ― 2.54) | 1.81(0.85 ― 3.14) | 2.55(1.74 ― 3.75) | 2.40(-0.33 ― 4.17) | 2.00(-0.59 ― 4.95) |
| Food worry (x_16_) | -1.02(-2.43 ― 0.46) | -2.21(-3.53 ― -0.95) | -3.42(-4.48 ― -2.54) | -3.37(-4.20 ― -2.47) | -3.61(-4.44 ― -2.41) | -3.18(-4.48 ― -2.02) | -2.64(-4.49 ― -0.76) | -3.06(-5.33 ― -0.11) |
| Get Health Assistance (x_17_) | 0.63(-0.94 ― 1.91) | 0.25(-0.57 ― 1.68) | 1.28(0.22 ― 2.12) | 0.81(0.18 ― 1.66) | 1.46(0.21 ― 2.38) | 3.02(1.78 ― 3.90) | 3.31(1.66 ― 5.28) | 3.05(0.74 ― 4.82) |
| Health problem (x_18_) | -0.30(-1.89 ― 0.81) | -0.76(-2.06 ― 0.40) | -1.05(-1.84 ― 0.17) | -0.73(-1.54 ― -0.14) | -1.38(-2.39 ― -0.29) | -2.61(-3.41 ― -1.41) | -2.72(-4.96 ― -0.71) | -2.77(-4.32 ― -0.19) |
| Household size (x_19_) | 0.90(-0.12 ― 1.66) | 1.47(0.24 ― 2.25) | 0.93(0.24 ― 1.86) | 1.14(0.63 ― 1.65) | 1.06(0.19 ― 1.89) | 2.01(1.02 ― 2.61) | 1.79(0.69 ― 2.92) | 1.53(0.08 ― 3.16) |
| Small size land ownership (x_20_) | -1.42(-3.32 ― 0.55) | -0.74(-2.37 ― 0.75) | -1.37(-2.92 ― -0.12) | -1.96(-3.07 ― -0.74) | -0.96(-2.19 ― 0.39) | -1.82(-3.48 ― -0.46) | -2.21(-4.87 ― -0.10) | -2.84(-6.88 ― -0.39) |
| Number of Livestock (x_21_) | 0.03(0.03 ― 0.04) | 0.03(0.02 ― 0.07) | 0.06(0.02 ― 0.12) | 0.07(0.04 ― 0.11) | 0.08(0.02 ― 0.14) | 0.13(0.04 ― 0.19) | 0.12(0.03 ― 0.20) | 0.04(-0.03 ― 0.18) |
| Meals eaten by Childe (x_22_) | 0.04(-0.36 ― 0.57) | 0.09(-0.26 ― 0.60) | 0.18(-0.17 ― 0.57) | -0.02(-0.22 ― 0.28) | -0.10(-0.51 ― 0.29) | -0.31(-0.69 ― 0.19) | -0.45(-0.96 ― 0.09) | -0.33(-0.93 ― 0.37) |
| Meals eaten by young (x_23_) | 0.61(-0.38 ― 1.60) | 0.63(-0.02 ― 1.49) | 1.22(0.65 ― 1.75) | 1.18(0.44 ― 1.67) | 1.17(0.50 ― 1.95) | 1.33(0.30 ― 2.29) | 1.65(0.63 ― 2.76) | 2.46(0.95 ― 3.71) |
| Soil property related (x_24_) | 0.70(-0.47 ― 1.29) | 0.87(0.24 ― 1.50) | 0.87(0.48 ― 1.20) | 0.31(0.06 ― 0.61) | 0.25(-0.07 ― 0.56) | 0.44(0.08 ― 0.96) | 0.07(-0.57 ― 1.19) | 0.46(-0.52 ― 1.23) |
| Agro-ecological & distance from border related (x_25_) | -0.18(-0.90 ― 0.84) | 0.08(-0.71 ― 0.81) | -0.21(-0.80 ― 0.28) | -0.75(-1.12 ― -0.26) | -1.02(-1.58 ― -0.53) | -2.11(-2.89 ― -1.54) | -2.30(-3.33 ― -1.34) | -2.25(-3.55 ― -1.25) |
| Rainfall & greens related (x_26_) | -0.34(-1.07 ― 0.67) | -0.61(-1.47 ― 0.34) | -0.99(-1.65 ― -0.34) | -0.94(-1.43 ― -0.44) | -0.92(-1.50 ― -0.44) | -0.90(-1.51 ― -0.50) | -1.05(-2.22 ― -0.30) | -1.32(-2.73 ― -0.22) |
| Rooting conditions & workability related (x_27_) | -0.44(-0.99 ― 0.31) | -0.06(-0.59 ― 0.49) | 0.40(-0.05 ― 0.83) | 0.57(0.27 ― 0.88) | 0.33(-0.06 ― 0.77) | 0.12(-0.30 ― 0.72) | -0.05(-0.75 ― 0.75) | -0.62(-1.47 ― 0.14) |
| Distance from market, road & population center related (x_28_) | 1.42(0.68 ― 1.82) | 0.77(0.28 ― 1.18) | 0.10(-0.20 ― 0.43) | 0.07(-0.24 ― 0.25) | -0.20(-0.54 ― 0.12) | -0.67(-1.13 ― -0.31) | -1.08(-1.92 ― -0.16) | -0.87(-1.99 ― -0.27) |
| Terrain Roughness & wetness related (x_29_) | -0.12(-0.60 ― 0.58) | -0.07(-0.48 ― 0.46) | 0.34(-0.02 ― 0.72) | 0.42(0.18 ― 0.72) | 0.73(0.25 ― 1.08) | 0.84(0.33 ― 1.28) | 0.94(0.25 ― 1.94) | 1.30(0.27 ― 2.05) |
| Agricultural package related (x_30_) | 0.07(-0.63 ― 0.67) | 0.52(-0.31 ― 1.13) | 0.16(-0.39 ― 0.65) | 0.27(-0.18 ― 0.65) | 0.14(-0.28 ― 0.63) | -0.58(-1.07 ― -0.06) | -0.06(-1.06 ― 0.83) | 0.78(-0.27 ― 2.09) |
| Agricultural material related (x_31_) | 0.80(-0.03 ― 1.44) | 1.29(0.56 ― 1.87) | 0.60(0.09 ― 0.99) | 0.42(0.10 ― 0.75) | 0.43(0.00 ― 0.80) | 0.02(-0.48 ― 0.46) | 0.36(-0.44 ― 1.10) | 0.16(-1.09 ― 1.23) |
| Blanket & Bed related (x_32_) | 1.86(1.24 ― 2.31) | 1.98(1.26 ― 2.49) | 1.72(1.29 ― 2.04) | 1.87(1.52 ― 2.10) | 1.64(1.28 ― 2.03) | 1.73(1.32 ― 2.08) | 2.02(1.24 ― 2.55) | 1.69(0.90 ― 2.82) |
| Drinking Water (x_33_) | 0.44(-0.05 ― 0.97) | 0.24(-0.34 ― 0.80) | -0.04(-0.39 ― 0.33) | 0.18(-0.10 ― 0.46) | 0.35(-0.07 ― 0.65) | 0.84(0.43 ― 1.26) | 1.21(0.47 ― 1.76) | 1.47(0.62 ― 2.40) |
| Electronic & Furniture related (x_34_) | 1.92(1.34 ― 2.58) | 1.67(1.11 ― 2.21) | 1.68(1.25 ― 2.21) | 2.11(1.73 ― 2.44) | 2.47(2.10 ― 2.93) | 2.80(2.40 ― 3.34) | 3.03(2.44 ― 4.06) | 3.24(2.58 ― 4.13) |
| Housing Quality related (x_35_) | 3.49(2.95 ― 3.96) | 3.57(3.15 ― 4.13) | 3.43(2.97 ― 3.79) | 3.47(3.20 ― 3.76) | 3.62(3.21 ― 4.08) | 4.11(3.55 ― 4.51) | 4.46(3.84 ― 5.14) | 4.96(4.13 ― 5.89) |
| Information Source related (x_36_) | 2.53(1.87 ― 2.96) | 2.81(2.22 ― 3.19) | 2.86(2.49 ― 3.27) | 3.00(2.66 ― 3.41) | 3.18(2.76 ― 3.56) | 3.77(3.32 ― 4.20) | 5.02(4.15 ― 5.88) | 5.11(4.59 ― 6.20) |
| Irrigation and related (x_37_) | -0.76(-1.69 ― -0.11) | -0.45(-1.42 ― 0.51) | 0.15(-0.48 ― 0.72) | 0.14(-0.24 ― 0.58) | 0.01(-0.68 ― 0.52) | -0.37(-1.09 ― 0.40) | -0.16(-1.11 ― 0.73) | -0.81(-1.87 ― 0.26) |
| Non-agricultural Business related (x_38_) | 0.50(0.11 ― 1.01) | 0.80(0.21 ― 1.37) | 1.00(0.53 ― 1.40) | 0.98(0.70 ― 1.25) | 0.95(0.61 ― 1.46) | 1.52(1.12 ― 1.90) | 1.59(0.82 ― 2.24) | 1.85(0.87 ― 2.49) |
| Productive Asset related (x_39_) | -0.19(-0.56 ― -0.03) | -0.10(-0.62 ― 0.17) | -0.18(-0.43 ― 0.25) | -0.20(-0.43 ― -0.04) | -0.40(-0.73 ― 0.26) | -0.26(-0.55 ― 0.07) | -0.06(-0.98 ― 0.57) | 0.00(-1.22 ― 0.87) |
| PSNP (x_40_) | 0.22(-0.48 ― 0.66) | -0.07(-0.55 ― 0.35) | -0.35(-0.66 ― -0.01) | -0.42(-0.65 ― 0.00) | -0.22(-0.62 ― 0.15) | 0.13(-0.46 ― 0.62) | 0.36(-0.42 ― 0.93) | 0.22(-0.45 ― 1.20) |
| Sanitation related (x_41_) | 1.27(0.55 ― 1.75) | 0.81(0.45 ― 1.50) | 0.63(0.27 ― 1.01) | 0.86(0.54 ― 1.15) | 1.22(0.84 ― 1.62) | 1.61(1.20 ― 2.05) | 2.20(1.31 ― 3.07) | 3.22(2.41 ― 4.11) |
| Region (x_42_): [Amhara ] | 1.62(-2.03 ― 4.65) | 2.12(-2.01 ― 6.58) | 3.45(-0.54 ― 6.11) | 5.19(3.12 ― 7.99) | 5.01(3.07 ― 8.24) | 4.42(0.80 ― 8.55) | 8.61(2.56 ― 13.15) | 9.02(1.50 ― 15.26) |
| [B.Gumuz] | -0.57(-2.75 ― 1.79) | 0.89(-1.29 ― 2.55) | 2.78(1.45 ― 4.20) | 2.84(1.83 ― 4.12) | 3.14(1.86 ― 4.78) | 2.40(1.05 ― 4.01) | 3.13(-0.17 ― 5.27) | 2.19(-1.37 ― 5.81) |
| [Diredwa] | 2.12(-0.83 ― 4.51) | 3.23(0.37 ― 5.32) | 7.75(6.14 ― 9.42) | 9.17(8.00 ― 10.61) | 9.38(7.92 ― 11.36) | 10.16(8.68 ― 12.12) | 12.14(8.02 ― 14.57) | 12.18(8.06 ― 15.90) |
| [Gambelia] | 5.72(2.06 ― 10.21) | 8.81(5.41 ― 11.16) | 9.71(7.06 ― 12.28) | 11.38(9.28 ― 13.45) | 9.70(7.71 ― 11.75) | 5.85(3.36 ― 7.88) | 7.68(2.13 ― 10.40) | 9.51(3.73 ― 13.74) |
| [Harari] | -0.31(-3.13 ― 2.65) | -0.05(-3.70 ― 2.77) | 4.00(0.96 ― 6.64) | 3.48(2.13 ― 5.80) | 1.74(-0.62 ― 4.63) | -1.32(-3.55 ― 1.19) | -1.27(-6.30 ― 2.53) | -1.28(-7.16 ― 5.03) |
| [Oromia] | -5.03(-8.25 ― -1.90) | -4.08(-6.71 ― -2.22) | -2.82(-4.23 ― -1.17) | -2.31(-3.67 ― -0.50) | -1.78(-3.17 ― 0.26) | -0.58(-2.14 ― 1.64) | 2.40(-1.80 ― 5.60) | 1.91(-2.50 ― 6.14) |
| [SNNP] | 1.02(-3.08 ― 6.14) | 4.51(0.60 ― 8.71) | 7.98(5.47 ― 10.88) | 7.06(4.80 ― 9.66) | 4.97(2.55 ― 8.11) | 4.27(0.84 ― 6.91) | 2.77(-2.41 ― 7.44) | 1.95(-3.48 ― 9.00) |
| [Somalie] | -1.42(-4.06 ― 3.91) | 2.61(-2.10 ― 5.66) | 3.57(0.52 ― 5.64) | 4.97(2.61 ― 7.30) | 5.55(2.78 ― 8.42) | 7.19(4.21 ― 11.69) | 11.14(7.22 ― 17.60) | 13.63(7.82 ― 18.44) |
| [Tigray] | 3.97(-0.54 ― 6.03) | 4.63(0.79 ― 7.82) | 11.64(8.29 ― 13.55) | 11.44(9.28 ― 13.40) | 10.55(8.53 ― 13.20) | 9.21(6.19 ― 12.20) | 10.04(6.36 ― 13.15) | 10.90(6.59 ― 16.21) |
| year:Mixed Cropping | -0.29(-0.83 ― 0.33) | -0.25(-0.96 ― 0.22) | -0.71(-1.12 ― -0.22) | -0.50(-0.80 ― -0.11) | -0.42(-0.95 ― -0.01) | -0.80(-1.33 ― -0.28) | -0.36(-1.15 ― 0.37) | -0.71(-1.67 ― 0.15) |

Appendix Table 3: Full results for linear mixed model (LMM) and Longitudinal Quantile regression

| Estimates | LMM | q0.05 | q0.10 | q0.15 | q0.25 | q0.35 | q0.5 | q0.75 | q0.9 | q0.95 |
| --- | --- | --- | --- | --- | --- | --- | --- | --- | --- | --- |
| (Intercept) | -1434.22*** | -1437.3*** | -1421.9*** | -1429.9*** | -1432.5*** | -1438.5*** | -1439.1*** | -1440*** | -1425.6*** | -1437.2*** |
| Year (x_1_) | 0.74*** | 0.72*** | 0.72*** | 0.72*** | 0.74*** | 0.74*** | 0.74*** | 0.74*** | 0.74*** | 0.74*** |
| Mixed Cropping (x_2_) | 1176.34*** | 1258.1*** | 1259.7*** | 1261.7*** | 1259.2*** | 1258.2*** | 1258.5*** | 1259.8*** | 1260.2*** | 1259.5*** |
| Urban Vs Rural (x_3_) | 0.72 | 7.89*** | 4.23 | -3.94* | 0.85 | 0.27 | 2.06 | 4.09*** | 4.6** | 12.44*** |
| Sex of household head (x_4_) | 0.2 | 0.22 | 0.49 | -2.19* | -0.08 | 0.65 | -0.61 | 0.5 | 0.98 | 2.21 |
| Read & Write (x_5_) | 1.95*** | 4.31*** | 0.65 | 2.63** | 0.55 | -1.98* | 1.98*** | 2.59*** | 3.41*** | 7.31*** |
| Shock (x_6_) | -1.08*** | -3.04*** | -3.33*** | -3.09*** | -1.43* | -0.58 | -1.89*** | -0.98* | -0.66 | -0.16 |
| Fertilizer (x_7_) | 0.73 | 1.7 | -0.65 | -1.17 | -0.18 | 1.44** | 0.2 | 2.28*** | 2.24** | 5.42*** |
| Adult equivalence (x_8_) | -1.14** | -1.65** | -1.45 | -1.41** | -1.48** | -0.8 | -1.31** | -0.99* | -1.07** | -1.74** |
| Age of household head (x_9_) | -0.01 | -0.19*** | -0.08* | -0.04 | -0.07*** | -0.03* | -0.01 | -0.02 | 0 | 0.02 |
| Assistance indicator (x_10_) | 0.32 | 3.1** | 4.76** | 2.13 | 0 | 0.35 | 0.12 | 1.3 | 2.52 | -0.79 |
| Copping Strategy Index (x_11_) | -0.03** | -0.03 | -0.12 | -0.11 | -0.14** | -0.12*** | -0.06** | -0.05 | -0.05* | 0.04 |
| Dependency Ratio (x_12_) | -0.3** | -0.3 | -1.22** | -1.55*** | -1.05*** | -0.71*** | -0.45** | -0.1 | -0.16 | 0.89 |
| Eat the same diet (x_13_) | -1.26 | -0.64 | -7.58 | 1.86 | -2.22 | -1.15 | -2** | 1.14 | -3.8*** | -1.28 |
| Employed (x_14_) | 2.34*** | -2.75 | 3.38 | 2.35 | 3.14** | 3.21*** | 3.91*** | 4.65*** | 5.35*** | -0.62 |
| Farm Type (x_15_):[Livestock] | 2.63*** | 15.84*** | 4.65 | 2.54 | 5.9** | 6.44*** | 8.82*** | 8.87*** | 6.6** | 13.32*** |
| Farm Type (x_15_) :[Both farms] | 1.31** | 2.74 | -0.79 | -1.09 | 1.33 | 1.07 | 1.77*** | 4.24*** | 2.88* | 1.89 |
| Food worry (x_16_) | -2.7*** | -0.59 | 2.74 | 3.64* | -1.43 | -2.02** | -0.96 | -0.4 | -0.69 | 0.04 |
| Get Health Assistance (x_17_) | 1.83*** | 2.95** | 0.5 | 2.04 | 1.84* | 1.2 | 1.03 | 1.81** | 2.43* | 5.07*** |
| Health problem (x_18_) | -1.36*** | 1.8 | -0.63 | -0.39 | -1.79* | -2.19** | -1.81*** | -0.64 | -0.55 | 1.86 |
| Household size (x_19_) | 1.51*** | 1.95*** | 1.78** | 2.07*** | 1.39*** | 1.04* | 1.5*** | 1.29*** | 1.6*** | 1.23** |
| Small size land ownership (x_20_) | -2.36*** | -4.2** | -3.86* | -5.92*** | -3.78*** | -2.74** | -2.61*** | -0.96 | -2.17 | -2.18 |
| Number of Livestock (x_21_) | 0.05*** | 0.05 | 0.08 | 0.05 | 0.06 | 0.02 | 0.06* | 0.08** | 0.06 | 0.01 |
| Meals eaten by Childe (x_22_) | -0.13 | 0.28 | 0.03 | -0.07 | -0.82*** | -0.44 | -0.25 | 0.13 | -0.18 | -0.3 |
| Meals eaten by young (x_23_) | 1.04*** | 1.39 | 0.69 | 2.24*** | 0.66 | 0.88 | 1.22*** | 1.07** | 1.69** | 1.1 |
| Soil property related (x_24_) | 0.62*** | 2.39*** | 0.75 | 0.39 | 0.85* | 0.64* | 0.4 | 1.44** | 1.49** | 1.55 |
| Agro-ecological & distance from border related (x_25_) | -1.11*** | 0.78 | 0.25 | 0.88 | 0.71 | 0.24 | -0.91*** | -0.95** | -0.52 | -2.68*** |
| Rainfall & greens related (x_26_) | -1*** | -0.75 | -0.71 | -1.71** | -1.4** | -0.86** | -1.15*** | -1.18** | -1.77** | -1.3* |
| Rooting conditions & workability related (x_27_) | 0.11 | -1.42* | 0.15 | 0.11 | -0.19 | 0.2 | 0.03 | -0.31 | -0.48 | -0.24 |
| Distance from market, road & population center related (x_28_) | -0.16 | 1.83** | 0.52 | 0.74 | 0.49 | 0.52 | -0.41 | -0.11 | -0.03 | -0.7 |
| Terrain Roughness & wetness related (x_29_) | 0.51** | 0.52 | 0.5 | 0.31 | 0.18 | 0.42 | 0.42 | 0.47 | 0.56 | -0.11 |
| Agricultural package related (x_30_) | 0.23 | 0.99 | 1.14 | 0.78 | 1.41*** | 0.44 | 1.08*** | 0.7* | 0.65* | 0.7 |
| Agricultural material related (x_31_) | 0.51** | 0.31 | 0.33 | 0.18 | 0.97** | 0.14 | 0.49* | 0.05 | 0.54 | 0.99* |
| Blanket & Bed related (x_32_) | 1.55*** | 1.65*** | 1.83*** | 1.45*** | 1.8*** | 1.55*** | 1.61*** | 1.87*** | 1.79*** | 2.56*** |
| Drinking Water (x_33_) | 0.57*** | -0.04 | -0.83 | -0.73 | -0.09 | 0 | 0.41 | 0.15 | 0.47 | 1.28** |
| Electronic & Furniture related (x_34_) | 2.22*** | 2.41*** | 1.15* | 2.28*** | 1.7*** | 2.36*** | 2*** | 2.79*** | 3.14*** | 5.78*** |
| Housing Quality related (x_35_) | 3.61*** | 2.56*** | 2.24*** | 2.55*** | 3.31*** | 3.51*** | 3.26*** | 3.25*** | 3.55*** | 3.45*** |
| Information Source related (x_36_) | 3.26*** | 1.96*** | 2.21*** | 1.78*** | 2.25*** | 3.08*** | 3.13*** | 3.42*** | 4.2*** | 3.27*** |
| Irrigation and related (x_37_) | -0.18 | -0.02 | 0.74 | 1.98** | 1.67*** | 0.01 | -0.15 | -0.15 | -0.48 | 0.66 |
| Non-agricultural Business related (x_38_) | 1.07*** | -1.04 | 0.54 | 0.27 | 0.67 | 0.78*** | 0.9*** | 0.84** | 0.47 | 0.26 |
| Productive Asset related (x_39_) | -0.11 | 0.38 | -0.2 | -1.18** | -0.32 | -0.51* | -0.09 | -0.06 | 0.25 | 1.23 |
| PSNP (x_40_) | -0.22 | -0.71 | -0.16 | -0.85* | -0.7* | -0.57 | -0.18 | -0.06 | -0.74* | 0.41 |
| Sanitation related (x_41_) | 1.32*** | 0.87 | 0.57 | 0.48 | 0.72* | 1.19*** | 1.32*** | 1.43*** | 1.55*** | 3.29*** |
| Region (x_42_): [Amhara ] | -2.24 | 8.02 | 7.75** | 5.5** | -1.42 | -1.88 | -0.03 | 2.86 | 1.17 | 6.84** |
| [B.Gumuz] | -3.4* | 9.96 | 12.53** | 15.77*** | 10.38*** | 6.15** | 4.67* | 5.37 | 9.2 | 16.44*** |
| [Diredwa] | 4.74*** | 24.39*** | 23.43*** | 22*** | 12.98*** | 15.19*** | 12.89*** | 16.27*** | 22.75*** | 26.72*** |
| [Gambelia] | 0.17 | 16.51** | 18.52*** | 19.46*** | 15.19*** | 12.07*** | 8.51*** | 11.94** | 13.92** | 22.55*** |
| [Harari] | 1.94 | 16.03** | 20.08*** | 16.39*** | 9.7*** | 11.25*** | 9.61*** | 15.4*** | 19.82*** | 25.9*** |
| [Oromia] | 4.07** | 10.67 | 15.04*** | 9.85*** | 5.08** | 4.75** | 6.58*** | 9.68*** | 9.99*** | 15.75*** |
| [SNNP] | -6.31*** | 1.04 | 4.68 | 2.15 | -6.84*** | -6.17*** | -4.37** | -1.44 | -0.88 | 3.51 |
| [Somalie] | 3.86*** | 16.96*** | 13.8*** | 9.75*** | 7.86*** | 8.16*** | 8.09*** | 10.98*** | 7.96* | 19.82*** |
| [Tigray] | -4.41*** | 4.76 | 8.86** | 2.48 | -1.55 | -2.84* | -2.37 | 3.14 | 5.51 | 13.37*** |
| year:Mixed Cropping | -0.58*** | -0.62*** | -0.62*** | -0.63*** | -0.63*** | -0.63*** | -0.62*** | -0.62*** | -0.63*** | -0.62*** |
| AIC | | 101896 | 100833 | 100635 | 99616 | 99189 | 98979 | 100717 | 102742 | 104759 |
| Log-likelihood | | -50893 | -50362 | -50263 | -49753 | -49539 | -49434 | -50304 | -51316 | -52324 |
| Covariance matrix of the random effects: (Individual specific variability, $\sigma_{\gamma_{i}}^{2}$) | | 125.6 | 105.9 | 130.8 | 108.3 | 81.44 | 70.14 | 108.9 | 165.7 | 190.6 |
| Residual scale parameter:(standard deviation,$\sqrt{\sigma^{2}}$ ) | | 1.099 (22.01) | 2.087 (21) | 2.968 (20.09) | 4.397 (18.54) | 5.391 (17.49) | 5.959 (16.85) | 4.624 (19.5) | 2.205 (22.18) | 1.249 (25.01) |
| ICC= $\frac{\sigma_{\gamma_{i}}^{2}}{\sigma_{\gamma_{i}}^{2}+\sigma^{2}}$ | | 0.21 | 0.19 | 0.24 | 0.24 | 0.21 | 0.20 | 0.22 | 0.25 | 0.23 |
| Significances: “***” for 99% , “**” for 95% & “*” for 90% | | | | | | | | | | |

^*^PSNP : Productive Safety nets Program

Appendix Table 4: Principal Component analysis for clusters of quantiles of Food security score derived by significance of major effects

| Quantile | PCA1 | PCA2 | PCA3 |
| --- | --- | --- | --- |
| q0.85 | 0.834 | 0.124 | 0.274 |
| q0.9 | 0.833 | -0.149 | -0.042 |
| q0.95 | 0.651 | 0.137 | -0.437 |
| q0.75 | 0.573 | -0.403 | -0.408 |
| q0.05 | 0.343 | 0.792 | -0.03 |
| q0.15 | -0.07 | 0.754 | -0.295 |
| q0.1 | -0.419 | 0.748 | 0.194 |
| q0.35 | 0.126 | -0.069 | 0.863 |
| q0.25 | -0.231 | 0.334 | 0.607 |
| q0.5 | -0.023 | -0.134 | 0.26 |
| Extraction Method: Principal Component Analysis with 63.6% of the variance considered or explained by the factors | | | |
| Rotation Method: Varimax with Kaiser Normalization. | | | |

Appendix Table 5: Code table for significance of major effects for longitudinal quantile regression model

| Major significant Factors | q0.05 | q0.1 | q0.15 | q0.25 | q0.35 | q0.5 | q0.75 | q0.85 | q0.9 | q0.95 |
| --- | --- | --- | --- | --- | --- | --- | --- | --- | --- | --- |
| Urban | 1 | 0 | 1 | 0 | 0 | 0 | 1 | 1 | 1 | 1 |
| Read & Write | 1 | 0 | 1 | 0 | 1 | 1 | 1 | 1 | 1 | 1 |
| Shock | 1 | 1 | 1 | 1 | 0 | 1 | 1 | 0 | 0 | 0 |
| Fertilizer | 0 | 0 | 0 | 0 | 0 | 0 | 1 | 1 | 1 | 1 |
| Adult equivalence | 1 | 0 | 1 | 1 | 0 | 1 | 1 | 1 | 1 | 1 |
| Age of household head | 1 | 1 | 0 | 1 | 1 | 0 | 0 | 1 | 0 | 0 |
| Copping strategy Index | 0 | 0 | 0 | 1 | 1 | 1 | 0 | 1 | 1 | 0 |
| Dependency Ratio | 0 | 1 | 1 | 1 | 1 | 1 | 0 | 0 | 0 | 0 |
| Employed | 0 | 0 | 0 | 1 | 1 | 1 | 1 | 1 | 1 | 0 |
| Farm Type : Livestock | 1 | 0 | 0 | 1 | 1 | 1 | 1 | 1 | 1 | 1 |
| Farm Type: Both farms | 0 | 0 | 0 | 0 | 0 | 1 | 1 | 1 | 1 | 0 |
| Health problem | 0 | 0 | 0 | 1 | 1 | 1 | 0 | 0 | 0 | 0 |
| Household size | 1 | 1 | 1 | 1 | 0 | 1 | 0 | 1 | 1 | 1 |
| Small size land ownership | 1 | 1 | 1 | 1 | 1 | 1 | 0 | 1 | 0 | 0 |
| Geo_PCA1 | 1 | 0 | 0 | 1 | 1 | 0 | 1 | 1 | 1 | 0 |
| Geo_PCA2 | 0 | 0 | 0 | 0 | 0 | 1 | 1 | 0 | 0 | 1 |
| Geo_PCA3 | 0 | 0 | 1 | 1 | 1 | 1 | 1 | 1 | 1 | 1 |
| PCA for Agricultural package | 0 | 0 | 0 | 1 | 0 | 1 | 1 | 0 | 1 | 0 |
| PCA for irrigation and related | 0 | 0 | 1 | 1 | 0 | 0 | 0 | 0 | 0 | 0 |
| PCA for non-agricultural Business | 0 | 0 | 0 | 0 | 1 | 1 | 1 | 1 | 0 | 0 |
| PCA for Sanitation | 0 | 0 | 0 | 1 | 1 | 1 | 1 | 1 | 1 | 1 |

Appendix Figure
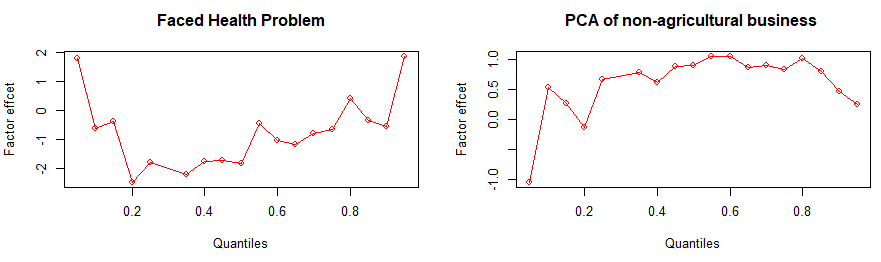

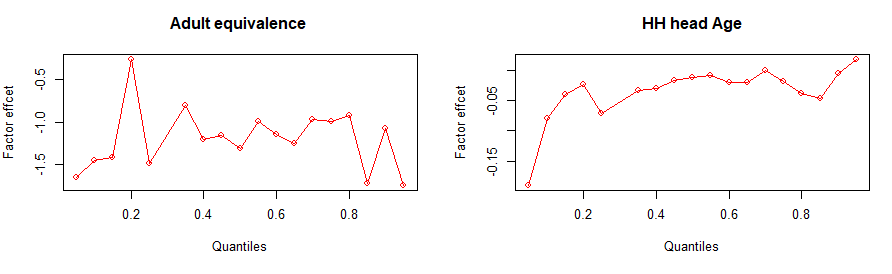


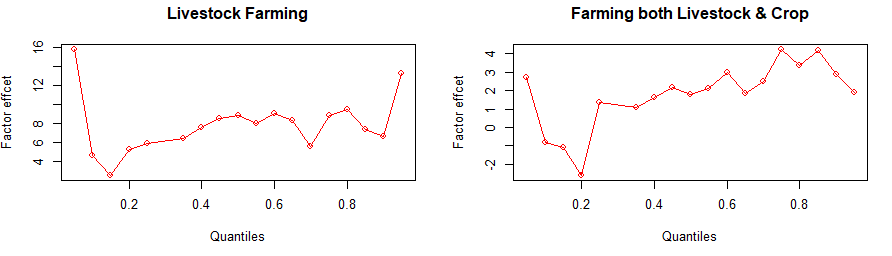


Appendix Figure 1: Distributional plot of longitudinal quantile regression coefficients estimate for Socio-economic & Agricultural factors.


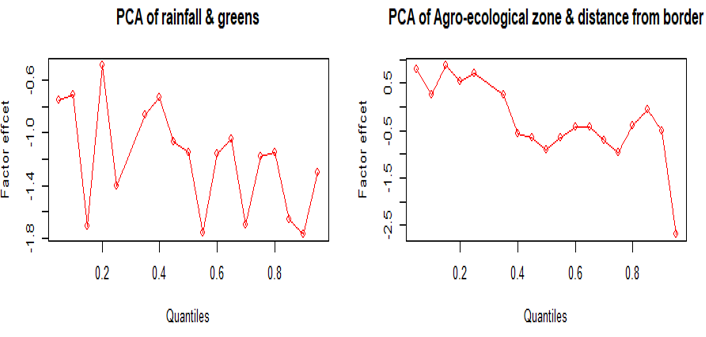

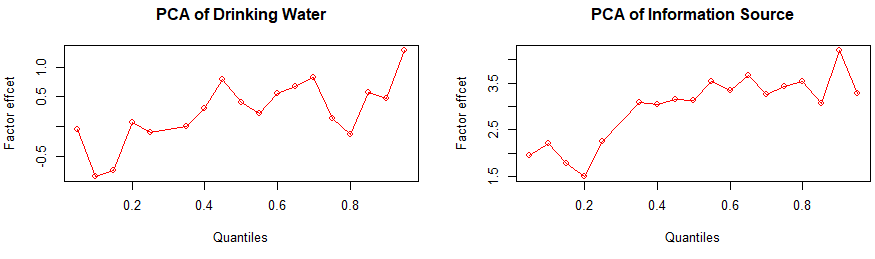

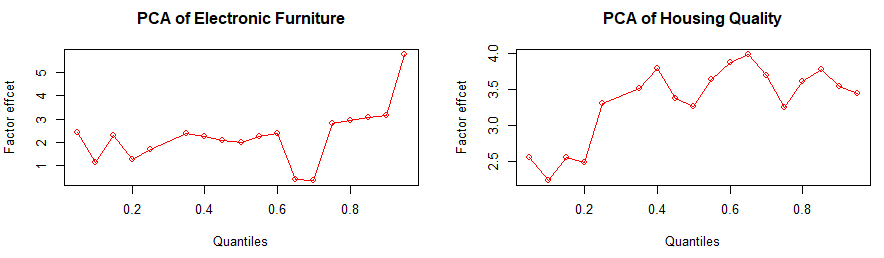


Appendix Figure 2: Distributional plot of longitudinal quantile regression coefficients estimate for Geographic & Asset components.


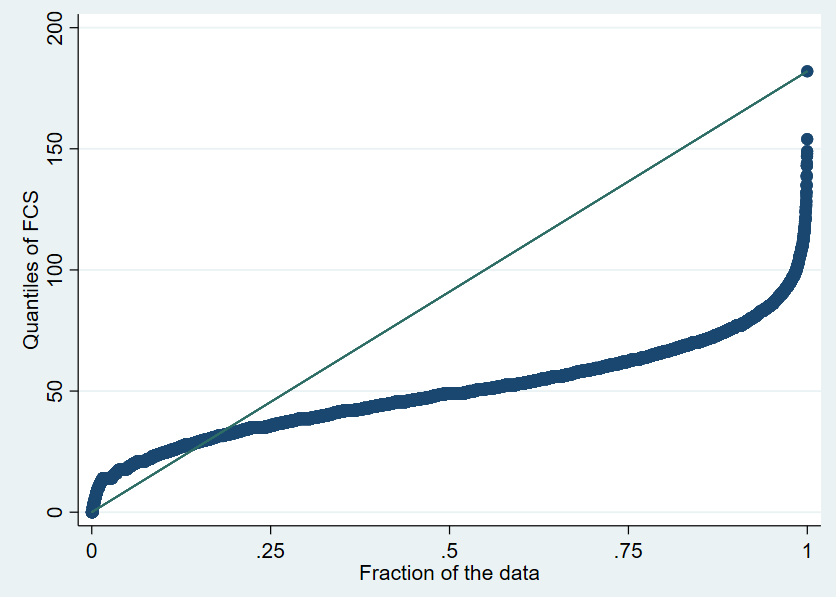


Appendix Figure 3: Longitudinal food security score quantiles plot
